# From Chihuahua to Saint-Bernard: how did digestion and microbiota evolve with dog sizes

**DOI:** 10.7150/ijbs.72770

**Published:** 2022-08-01

**Authors:** Charlotte Deschamps, Delphine Humbert, Jürgen Zentek, Sylvain Denis, Nathalie Priymenko, Emmanuelle Apper, Stéphanie Blanquet-Diot

**Affiliations:** 1 Université Clermont Auvergne, UMR 454 MEDIS UCA-INRAE, Clermont-Ferrand, France; 2 Lallemand Animal Nutrition, Blagnac, France; 3 Dômes Pharma, Pont-du-Château, France; 4 Institute of Animal Nutrition, Freie Universität Berlin, Königin-Luise-Strasse 49, Berlin, Germany; 5 Toxalim (Research Center in Food Toxicology), University of Toulouse, INRAE, ENVT, INP-Purpan, UPS, Toulouse, France

**Keywords:** canine, digestive physiology, gut microbiota, petfood, veterinary products

## Abstract

Health and well-being of dogs are of paramount importance to their owners. Digestion plays a key role in dog health, involving physicochemical, mechanical and microbial actors. However, decades of breeding selection led to various dog sizes associated with different digestive physiology and disease sensitivity. Developing new products requires the consideration of all the multi-faceted aspects of canine digestion, the evaluation of food digestibility, drug release and absorption in the gut. This review paper provides an exhaustive literature survey on canine digestive physiology, focusing on size effect on anatomy and digestive parameters, with graphical representation of data classified as “small”, “medium” and “large” dogs. Despite the huge variability between protocols and animals, interesting size effects on gastrointestinal physiology were highlighted, mainly related to the colonic compartment. Colonic measurements, transit time permeability, fibre degradation, faecal short-chain fatty acid concentration and faecal water content increase while faecal bile acid concentration decreases with body size. A negative correlation between body weight and *Proteobacteria* relative abundance was observed suggesting an effect of dog body size on faecal microbiota. This paper gathers helpful *in* vivo data for academics and industrials and supports the development of new food and pharma products to move towards canine personalized nutrition and health.

## Introduction

*Canis lupus familiaris*, also known as the domesticated dogs, belong to the *Canidae* family like the grey wolf (*Canis lupus*) and the dingo, a domestic dog returned to the wild. Descending from the grey wolf, dogs might have been the first animal domesticated by humans around 20.000 to 40.000 years ago 1. Dogs were initially strict carnivores, but during the agricultural revolution they probably acquired the ability to digest starch and became facultative carnivores. Genes playing key roles in starch digestion (i.e. encoding for pancreatic amylase, membrane-bound intestinal maltase-glucoamylase and gene involved in glucose uptake) were selected during dog domestication 2. Depending on their usefulness for humans, the *Canis lupus familiaris* subspecies have differentiated slowly, with the development of new canine species designated for specific tasks, such as herd protection (Mastiff), hunting (Pointer), cold hardiness (Siberian husky) or companion (Pekinese). Nowadays, the canine species includes approximatively 400 breeds with high size variability and weight ranging from 1 kg for a Chihuahua to 100 kg for a Saint-Bernard 3. Dogs now occupy a full place in many families. Their health and well-being are therefore of paramount importance to their owners, to the extent that 7 % of French dogs have their own health insurance against 30 % of dogs homed in the United Kingdom and 80 % of dogs homed in Sweden (SantéVet/Ipsos, 2018). Digestion, a complex process involving many physicochemical, mechanical, and microbial mechanisms, is a key parameter in dog health. In particular, gut microbiota and its involvement in canine nutrition and health have increasingly been studied during the last decade. Developing new food or pharma products needs to consider all these multi-faceted aspects of canine digestion, to answer important questions such as food digestibility, micronutrient bioaccessibility, probiotic survival and activity, or drug release and absorption in the gut. Petfood manufacturers and veterinary companies aim to develop personalized ranges adapted to size (e.g. long-term growth of large breeds puppies, poor digestive tolerance and gastric dilatation volvulus for large dogs) or to address certain breed predispositions such as obesity in Labrador Retrievers or enteropathies in Terriers 4-7. Nevertheless, the impact of dog size or breed on digestive parameters remains poorly described despite its full interest in canine nutrition and health.

This review paper provides for the first time an exhaustive survey of the literature on the impact of body size on dog's digestive physiology, in the entire gut from mouth to colon and feces, by gathering digestive anatomy, physicochemical parameters and gut microbiota variations. Relevant studies were identified, and information extracted regarding involved dogs (i.e. number of dogs, age, weight, breed, sex, reproduction state, living environment), nutrition (i.e. food type, feeding frequency, food's principal components) and analysis methods. Only *in vivo* studies on healthy adult dogs, fed with dry food or ingesting water were included. Here, canine body sizes were classified into three groups: “small” under 10 kg, “medium” between 10-30 kg and “large” up to 30 kg according to usual practice of main petfood suppliers. Then, the selected data were analyzed according to dog sizes and clarified through graphical representations to highlight a potential size effect on digestive parameters.

## General observations of canine digestion and associated organs

External morphological differences observed between extreme dog sizes such as Chihuahua and Saint-Bernard obviously reveal internal anatomical modifications. The canine mature digestive tract length can represent 2.8 % to 7 % of the total body weight (BW), in a 60 kg and a 5 kg dog, respectively 8. Since gastrointestinal tract (GIT) absolute length (in centimeters) is a reflect of dog height at the shoulder with a 6:1 ratio 9, it leads to the question: how does the size of dog impact digestive anatomy? Canine digestive anatomy is adapted to their facultative carnivorous diet (i.e. high-protein and high-fat diet) with a short and simple digestive tract. Digestion starts in the mouth with mastication process, helped by saliva. After swallowing, food boluses are transported through the esophagus into the stomach which is a J-shaped organ of glandular type, characterized by three anatomical compartments (i.e. fundus, corps and antrum) leading to the pylorus sphincter 10. Canine gastric mucosal cells secrete hydrochloric acid (HCl), pepsin and lipase, which makes stomach essential in protein and lipid digestion. Canine stomach has a high dilatation capacity, varying from a maximal volume of 0.5 L for small dogs to 8 L in large dogs, which corresponds to the extreme quantity of food that a dog can ingest 10. Digestion continues along the small intestine which is distributed as 10 % length for duodenum, 85 % for jejunum and 5 % for ileum 10,11. Small intestine length measured *post mortem* is positively correlated (Pearson correlation of 0.672) to canine BW (from 240 cm for a 5 kg to 640 cm for a 33 kg dog), as well as small intestine width (weaker correlation, R^2^ = 0.36) 11. Canine small intestine, together with peripheral organs such as pancreas and liver, have a key role in canine digestion process. Pancreas produces pancreatic juice delivered into duodenum and associated with protein, carbohydrate and lipid digestion. Liver, coupled with gallbladder, has a central role in lipid digestion through bile acid (BA) production and induction of increased intestinal peristalsis 12. Small intestine is also a central player in nutrient absorption, allowed by the presence of microvilli at the surface of enterocytes. When measuring intestinal wall thickness at different levels of the GIT (descending duodenum, proximal and distal jejunum, proximal and distal ileum), higher values were observed for male dogs compared to female (except for distal ileum) but no correlation was found with dog sizes whatever the intestinal compartment 13. Regarding small intestinal villus length, an old study from 1978 showed no correlation between dog weight and mucosal dimensions 14. In adult dogs from various sizes, duodenal villus length was 722 ± 170 µm 15. Jejunal villi were longer in small dogs like Pomeranian and Fox Terrier (900 μm) than in medium ones such as Newfoundland (500 µm) 16. Lastly, ileal villus length was measured in medium size Greyhound female and values around 796-823 µm were found 17. Canine large intestine measures around 20-80 cm with 2-3 cm diameter in medium dogs 10. The three parts of the canine colon (i.e. ascending, transverse and descending) are not so well defined when compared to humans, with the particularity to be non-sacculated and devoid of sigmoid colon 10. Ascending colon represents in medium size dog 20% of the colon length, while transverse and descending correspond to 30 % and 50 %, respectively. The two first parts are used for transport, electrolyte and water modification as well as for bacterial fermentation and storage areas, while descending colon mainly functions as conduit ending with rectum. Canine large intestine is involved in water and electrolyte absorption but also degradation of residual nutrients thanks to the fermentation activity of resident microorganisms called gut microbiota. Large intestine total length appears to vary according to dog's BW, from 32 cm for Miniature Poodles to 99 cm for Great Danes 18. Volume and surface are also increased from Miniature Poodle to Great Dane (volume of 92 *versus* 2106 cm^3^, surface of 191 *versus* 1612 cm^2^). As the large intestine length increases with BW, the same positive relation is observed for absorption surface with a higher number of villi in large compared to small dogs 18. Colonic crypts length was around 500-600 µm but without correlation with dog size 16. To conclude, scarce anatomy data (only five publications) evidenced morphological differences depending on dog's BW (mainly related to the colonic compartment), even if important parameters have not been evaluated such as gastric wall thickness, intestinal microvilli characteristics (i.e. length or number) or peripheral organs anatomy and functions. Variations in digestive anatomy can obviously affect physicochemical parameters such as pH, digestive secretions and transit time, and consequently gut microbiota.

## Methods of literature research

Our literature search was performed using *PubMed* (https://pubmed.ncbi.nlm.nih.gov) and *Google Scholar* (https://scholar.google.fr) with the key words “dog” OR “canine” AND “stomach”, “small intestine”, “intestine”, “duodenum”, “jejunum”, “ileum”, “ileal”, “colonic”, “large intestine”, “rectum”, “feces”, AND “anatomy”, “digestion”, “pH”, “enzyme”, “digestive secretion”, “digestibility”, “permeability”, “absorption”, “microbiota”, “bile acids”, “transit time”, “fatty acids”, “fermentation”, “gas”, “mucus” in all available years. The online database search was last performed in January 2022 on titles, abstracts and key words including original articles, reviews, thesis, and books. Relevant studies were identified after consultation of the main text, figures, and supplementary materials. Information regarding involved dogs (i.e. number of dogs, age, weight, breed, sex, reproduction state, living environment), diet (i.e. type of food, feeding frequency, composition of food), health (i.e. healthy dogs only) and analysis methods were collected. Only *in vivo* studies on adult dogs, fed with dry food or ingesting water were included in the literature survey.

We found a total of 163 studies, including 87 providing information on a single dog size, with only small dogs involved in 7 publications, only medium dogs in 71 and only large dogs in 9 (**Figure S1**). The three dog sizes (i.e. small, medium and large dogs) were compared together in 8 additional studies. In the remaining 68 studies, 45 integrated dogs without specifying their characteristics and other 22 included different sizes of dogs but didn't discriminate them in their analysis (both classed in the “unclassified” group). Concerning publication date, 40 studies were performed over 30 years, 76 studies have been done between 5 and 30 years ago and 47 were performed in the last 5 years. Only 10 studies were directly targeting the influence of dog size on canine digestive physiology.

## Impact of body size on digestive physicochemical parameters

### Gastrointestinal pH

**Figure 1:**
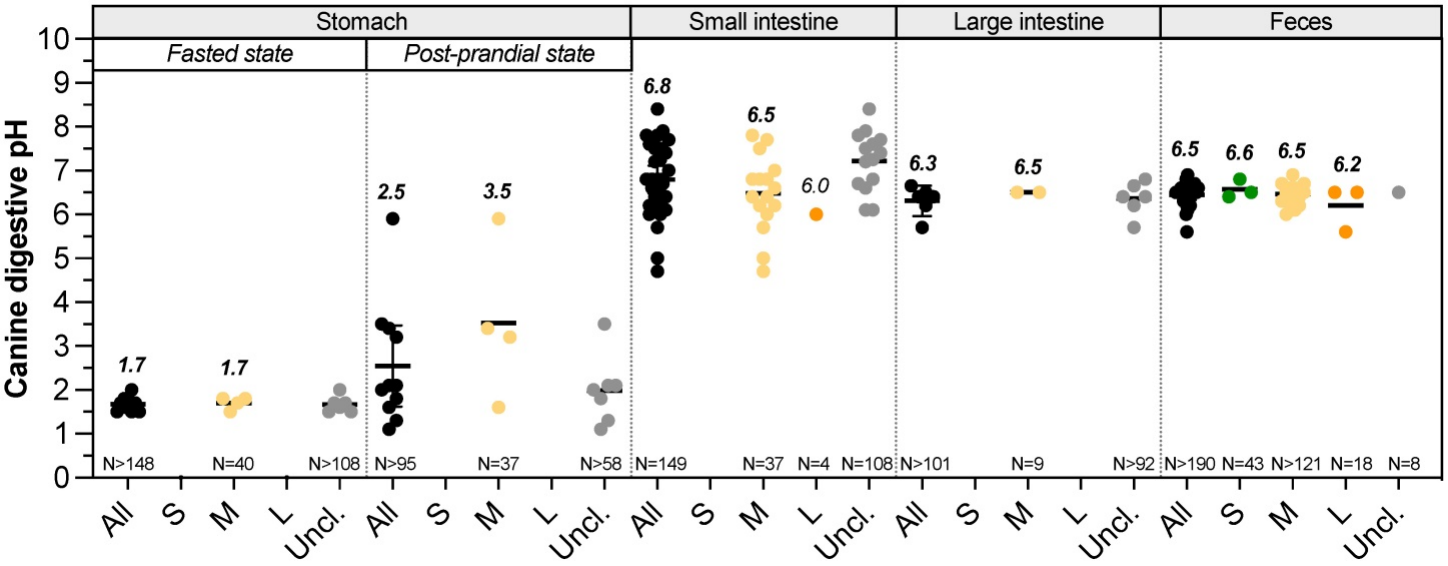
** Impact of dog sizes on pH values in all digestive compartments.** Results from studies measuring in dog's pH values in the stomach (under fasted or fed conditions), small intestine, large intestine and faeces are presented. Small dogs are plotted in green, medium dogs in yellow, large dogs in orange and unclassified dogs in grey. Raw data were pooled in “all” group (in black). Calculated median values are in italic bold, values for a single point in italic. Black bars represent 95% confidence intervals. The number of dogs involved in studies is indicated as “N=”

Gastrointestinal pH changes along the dog digestive tract (**Fig. 1**, **Table S1**). Mean salivary pH of medium dogs is around 7.3-7.8 and quickly decreases by 0.5 point with a stimulation using a piece of solid sugar on the tongue 19-21. In the stomach, the arrival of food bolus stimulates HCl production. This compartment shows the lowest pH value along the GIT, allowing dogs to partially digest bones 22 and putrescent meat and largely depends on feed status. However, due to the paucity of data, it remains difficult to know how BW affects gastric, small intestinal and colonic pH (**Fig. 1**, **Table S1**). To date, gastric pH has not been assessed in small and large dogs 23-26. Regarding medium dogs under fasted conditions, mean gastric pH of Beagles is around 1.5 (range 0.9-2.5), punctuated by occasional pH spikes with high frequency changes due to inter-individual variability 27. Those values measured in laboratory animals are in accordance with pH found in mixed-breed owner dogs 28. Small intestinal pH increases to value close to the neutrality because of the buffering capacity of pancreatic juice enriched in bicarbonate ions and bile 10. It also increases from the proximal to the distal parts, from 6.5 to 8 in medium size dogs 29. To date, there is no available study that investigates the influence of the dog size on duodenal and ileal pH 30. The few studies investigating the canine jejunal pH measured a mean pH of 6.8 and 6.0 for medium and large dogs, respectively 31,32. Only few studies investigated colonic pH using colonic cannula or wireless capsules, and once new, most of them do not discriminate dogs in terms of BW. Colonic pH is more acidic than the small intestine one, with mean values of 5-6.5 and 6.2, respectively for medium and large dogs, whereas there is no data concerning small dogs 29,33-35. Most of the time, colonic pH is estimated using faeces and there is no information on how pH varies depending on colonic sections. The average canine faecal pH values are in accordance with colonic pH, mainly around 6.4-6.6, as observed in **Fig. 1**. For small dog group, three studies used faeces of 43 dogs and pH values vary weakly from 6.4 to 6.8 36-38. There are also plenty of data on the faecal pH of medium (more than 121 dogs) and large (18 dogs) size dogs, with a pH range of 6-6.9 and 5.6-6.5, respectively 39-42. This is an accordance with some studies reporting that colonic and faecal pH of large dogs are more acidic than that of other size dogs fed with the same diet 18,43.

### Digestive secretion

**Enzymes**. First digestion step occurs in the oral cavity with salivary enzymes (**Table S2**). Numerous recent studies measured amylase activity in saliva of healthy dogs 44-49. Mean amylase activity varies from 26.5 to 37.3 UI/L of saliva in medium dogs according to literature (**Table S2).** One study involves 75 dogs from 8 to 42 kg (52 mixed breeds and 23 pure breeds) and measured 35.9 ± 41 UI/L amylase in saliva but results weren't discussed regarding dog sizes 48. Lactate dehydrogenase and adenosine deaminase activities were also quantified in saliva, without classification with canine BW 45,48,50. Gastric mucosa secretes gastric juice containing proteolytic (pepsin, chymosin) and lipolytic (lipase) enzymes 20,51. In laboratory Beagles, gastric juice volume output increases with meal viscosity, from a total of 37.2 mL secreted for a low viscosity to 190 mL for a high viscosity meal 52. Pancreatic juice, discharged in canine duodenum, has an alkaline pH (7.4-8.3). It contains amylase (2013 U/kg BW), lipase (9.8-33.3 mL 0.05 N NaOH/mL -no longer used unit of measure), phospholipases, cholesterases, proteases (old value of 407.5-2440 mg tyrosin/mL -no longer used unit of measure) and nucleases, without further detailed information 12,53. Digestive secretions were mainly studied before 2000s, but values were not discriminated depending on dog sizes, and no study focuses on small and large dogs. However, enzymatic activities may vary according to the different diet composition (i.e. protein, lipid, fiber contents) adapted to each dog size.

**Figure 2:**
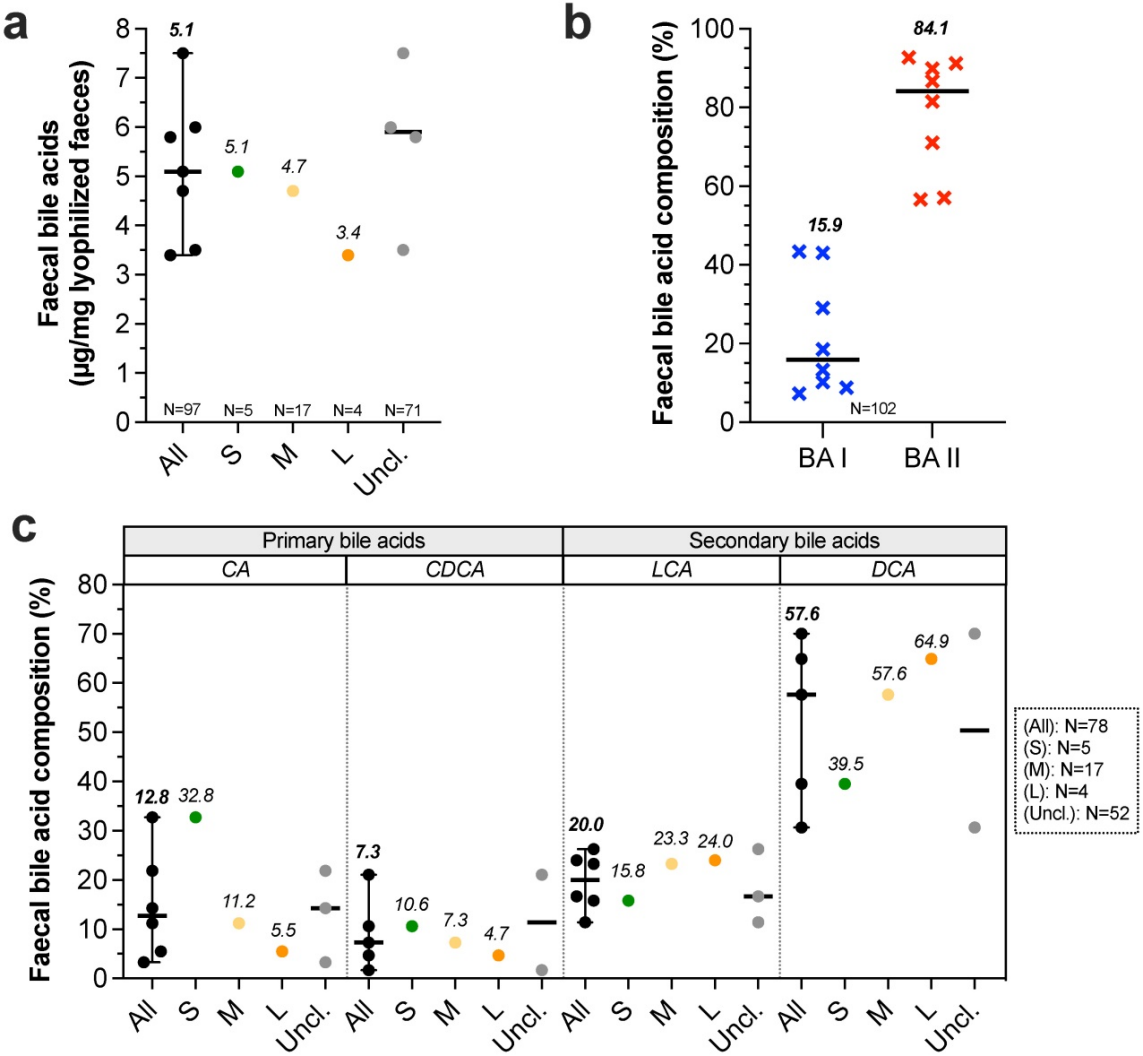
**Impact of dog sizes on faecal bile acids.** Results from studies in dog faeces quantifying total bile acids (BA) are represented in (a), further separated into primary (blue crosses) and secondary BA (red crosses) in (b). Detailed composition in cholic acid (CA), chenodeoxycholic acid (CDCA), lithocholic acid (LCA) and deoxycholic acid (DCA) is shown in (c). The same caption as used in **Fig. 1** was applied

** Bile salts**. Bile is produced by liver, partially stored in gallbladder then discharged to duodenum during postprandial phase, allowing stimulation of intestinal motility, intestinal lipids saponification and vitamins A, D, E and K absorption. In liver, primary BA such as cholic acid (CA) and chenodeoxycholic acid (CDCA) are synthesized from cholesterol and conjugated to taurine or glycine 54. Studies evaluating bile production in healthy dogs never discriminate dog sizes. Bile production was only evaluated in medium dogs and was found to be 29 mL/kg/24 h 55. Once reached gallbladder, bile is up to 10 fold more concentrated than in liver with a total concentration around 50 (40-90) mmol/L 10,54,56,57. Here, it contains up to 15 different BA but the three majors count for 99% of total pool, with 72.8% taurocholic acid, 20.3% taurodeoxycholic acid and 6.2% taurochenodeoxycholic acid 58. In the small intestine, BA are deconjugated by gut microbiota and converted into secondary BA. 95% of BA are reabsorbed in ileum, return into liver and the 5% remaining fraction crosses colon 56. Faecal BA concentrations were measured in three recent studies involving all dog sizes but curiously without BW distinction (**Fig. 2A, Table S2**). Authors found coherent results with concentrations ranged from 5.8 to 7.5 µg of total BA per mg of dry faeces 59-61. Another recent study evaluated faecal BA concentrations in 24 healthy dogs 62. After data retreatment (classification in size groups), small, medium and large dogs present respectively 5.1, 4.7 and 3.4 µg/mg of total BA per mg of dry faeces. This suggests a decrease in faecal BA concentrations with BW increase. Further analysis from 8 studies (**Fig. 2B**) indicates that relative percentages of faecal secondary BA (BA II: 84.9%) are higher than primary BA (BA I: 15.5%). Moreover, proportions of primary BA such as CA and CDCA seem to be inversely correlated to canine BW whereas the contrary is observed for secondary BA (only one study) (**Fig. 2C**). These results suggest that the microbiota activity, and notably the BA recycling, differs from small to large breed sizes.

**Mucins.** Mucins are produced by goblet cells all along the dog GIT 10. Mucus thickness has been evaluated only in gastric compartment and stomach presents a mucosa covering mucin-layer of 576 μm and 425 μm, respectively in the antrum and fundus 10,63,64. This mucin-layer allows protection of the epithelium against acidic pH of stomach and withstands bone fragments 65. Influence of dog size on mucin secretion and mucin-layer thickness whatever the digestive compartment has never been assessed.

### Nutrient digestibility

**Figure 3:**
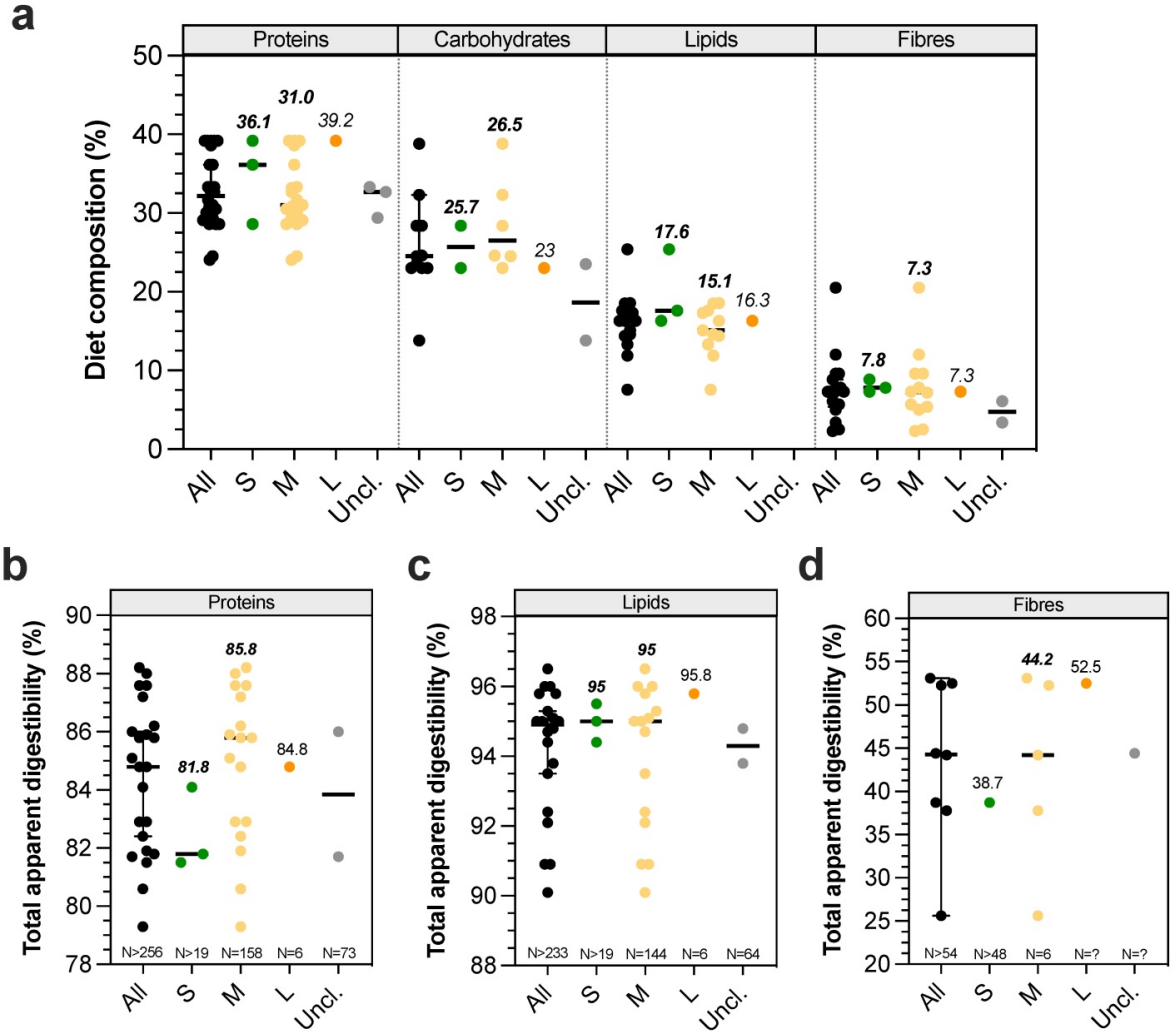
** Diet composition and impact of dog sizes on total apparent digestibility.** Nutritional composition of dry food diet used in canine studies is represented in (a). Results from studies investigating in dogs' total digestibility of proteins, lipids and fibres are presented in (b), (c) and (d), respectively. The same caption as used in **Fig. 1** was applied

Digestibility defines the degree to which organic matter is digested by an animal. Its measure provides a qualitative and quantitative indicator of food's quality, i.e. the more digestible a food is, the higher the proportion of absorbed nutrients will be. **Figure 3A** gives an overview of canine dry food composition in dogs according to BW. Digestibility performances can be evaluated in dogs by measuring ileal or total (in faeces) apparent digestibility of a tested diet, and standardized digestibility could be obtained by deducing endogen products such as enzymes or metabolites delivered from intestinal cell desquamation. As previously observed for physicochemical parameters, digestibility studies are mainly focused on medium dogs and there are only two publications on small 66,67 and one on large dogs 66 (**Table S3**). Due to their invasive nature, only 4 studies have been performed with ileal cannula (to measure ileal digestibility), including 3 on medium dogs 68-71. Lipid digestibility seems to be almost complete at the ileum level (i.e. 89.3-96.5%), with only around 3-5% increased digestibility when evaluating total digestibility in faecal samples. Ileal protein digestibility appears to be lower (51.3-76.2%), with higher variations certainly related to protein quality which largely influences this parameter 67,72-74. Surprisingly, the only study investigating total dietary fibre digestibility found an ileal digestibility of 17.8%, while according to their definition fibres are only degraded in the large intestine 68. Given the lack of data, it is impossible to conclude on a possible effect of dog BW on ileal nutrient digestibility. Total apparent protein (82-88%) and lipid (95-95.8%) digestibilities appear to be equal between different dog sizes, whatever the initial proportion of dietary proteins or lipids (**Fig. 3B-C**). In contrast, total apparent dietary fibre digestibility (**Fig. 3D**) appears to be higher in large than in small and medium dogs (52.5 ± 4% for Great Dane *versus* 39 ± 7.4% for Miniature poodle, and 26-38% for medium dogs) 42,75,76. Indeed, it seems that fibre digestibility would be quite similar between small and medium dogs, while it would be improved in large dogs. In addition, faecal apparent digestibility of dry matter, organic matter and gross energy appears to be significantly higher for large compared to small dogs 66. All in all, those results mean that the colonic fermentation seems to be more important in large than in medium and small breed size dogs.

### Intestinal absorption

**Permeability.** During digestion process, food is broken down into small soluble compounds (amino acids, fatty acids, monosaccharides, minerals and vitamins), able to be absorbed mainly through the villi-covered wall of the small intestine. Nutrient passage through the epithelial wall is modulated by intestinal permeability, which is the property of epithelium to allow some molecules to be absorbed passively or actively through mucosa while avoiding the passage of microorganisms and macromolecules. Lactulose to L-rhamnose or lactulose to sucralose urinary ratios could be used to monitor changes in canine small and large intestine permeability, respectively 77. A higher lactulose to L-rhamnose ratio is associated with a leakier small intestine, while a lower lactulose to L-rhamnose ratio indicates a higher colonic permeability. Using these methods, Weber *et al.* 78 observed an increased intestinal permeability in Giant Schnauzer and Great Danes (large dogs; lactulose to L-rhamnose ratio: 0.31) compared to small dogs (0.16), and Hernot *et al.* 77 found a higher colonic permeability in large dogs (lactulose to sucralose ratio: 0.35) than in small ones (0.51). Those differences could be related to modifications associated with dog size in intestinal area, pore size, frequency of tight junctions, differences in tightness of tight junctions or accessibility of luminal content to intestinal crypts 79. Of note, breed differences were particularly noticed with a higher colonic permeability in Great Danes, as previously described 80,81. An increased permeability could affect both nutrient, metabolite and electrolyte absorption but also microorganism's translocation. This may explain the weaker digestive tolerance of resistant-starch and higher digestive sensibility of large dogs compared to small ones, as discussed by Goudez et al. 82.

**Passive absorption.** Water, electrolytes and vitamins are absorbed through passive mechanisms in the small and large intestinal lumen. In healthy dogs, around 90% fluids crossing the colon are reabsorbed by mucosa 20,83. Meyer *et al*. 81 demonstrated that total faecal water increases with dog BW, but the percentage of free faecal water decreases. This is of high interest because an increase in free faecal water content is associated with a higher colonic water content that can in turn influence *in vivo* drug dissolution, in the case of poorly soluble drugs for which dissolution continues in the large intestine 43. Whereas small dogs tend to have drier stools, a tendency of poorer faecal consistency and higher water content is observed in larger dogs. Potassium and bicarbonate ions are secreted into the colonic lumen, whereas sodium and chloride ions are passively absorbed from luminal contents 83. Uptake of sodium ions creates an hypertonic environment next to crypts, generating an electrochemical gradient across colonic mucosa which drives water uptake from luminal contents by osmosis 83. Based on observation that large digestibility variations are observed within the same breed and between different breeds, Zentek and Meyer 80 compared mineral digestibility of four food types in Great Danes and Beagles. There was no breed difference for calcium, magnesium and phosphorous absorption (**Table S3**), while net colonic sodium absorption tended to be 9-23% lower in Great Danes compared to Beagles. These data were further supported by Weber *et al.* 36 describing an increase in sodium faecal content with an increase in BW (2.1 ± 0.7 g/kg DM in Miniature Poodle *versus* 6.1 ± 1.3 g/kg DM in Great Dane), traducing a lower sodium absorption by large dogs. Moreover, a reduction of colonic absorption of sodium has been particularly observed in Beagle, Labrador Retriever, Springer Spaniel and Münsterländer, suggesting a breed sensitivity 18. Besides, Neri *et al.* 84 reported a significantly greater faecal potassium concentration in large compared to smaller dogs. Independently of dog sizes, 90% vitamin D, 80-90% vitamin A, 40-90% vitamin K and 35-50% vitamin E are absorbed by passive absorption in the proximal small intestine 20.

**Active absorption.** Active absorption processes in the small intestine implicate co-transporters (e.g. glucose or sodium-dependent transports) and concerns monosaccharides from carbohydrate degradation and peptides from protein degradation. Thus, 95% of monosaccharides are absorbed in the duodenum and proximal jejunum 20, and 30% of amino acids and 70% of tripeptides are absorbed and assimilated in the proximal jejunum 12. Regarding lipids, 80% of fatty acids and monoglycerides are absorbed in form of micelles in the small intestine and resulting in chylomicrons that passed into the intestinal lymphatic capillary of villus by endocytosis. There is no available information on the influence of dog size on nutrient absorption. Moreover, the overall active transport capacity of small intestine has been assessed by examining urinary excretion ratio of D-xylose to 3-O-methyl-D-glucose 78. Non-significantly different ratios of 0.57, 0.58 and 0.59 for small, medium and large dogs respectively have been reported, suggesting that small intestinal active transport is relatively consistent between sizes.

### Mechanical digestion and gastrointestinal transit time

**Motility.** Canine gut motility was firstly evaluated using radiopaque markers, plastic beads or breath test. Recently, wireless motility capsule was developed to measure pressure, forces and gut contractions frequency. Using this method, Boscan *et al.* 85 observed in fed medium dogs a lower maximal amplitude contraction in the stomach compared to small intestine (52 mmHg *versus* 75 mmHg, respectively), coupled with higher gastric contraction frequency, with 3.7 contractions/min in the stomach *versus* 0.5 contraction/min in the small intestine. Another study involving dogs from different sizes observed similar tendency on maximal amplitude contraction (lower in stomach than in small intestine, with 0.2 *versus* 4.1 mmHg), but opposite results on frequency (0.8 in stomach *versus* 10.9 contractions/min in small intestine) 86. Moreover, in this study, large intestinal contraction frequency seems to be similar to the gastric ones (0.6 contraction/min). Authors also calculated a motility index defined as the area under the pressure curve and the higher motility index was observed in small intestine (306.2 compared to 20 in stomach and 76.1 in colon). Using wireless motility capsule, Farmer *et al.* 87 found that motility indexes were higher in large intestine (199 mmHg*second/min) compared to small intestine (134 mmHg*second/min) and stomach (55 mmHg*second/min) with a similar maximum of 3.7 contractions/min in gastric compartment 88. Lastly, no study has investigated how dog BW or size influences gut peristalsis.

**Figure 4:**
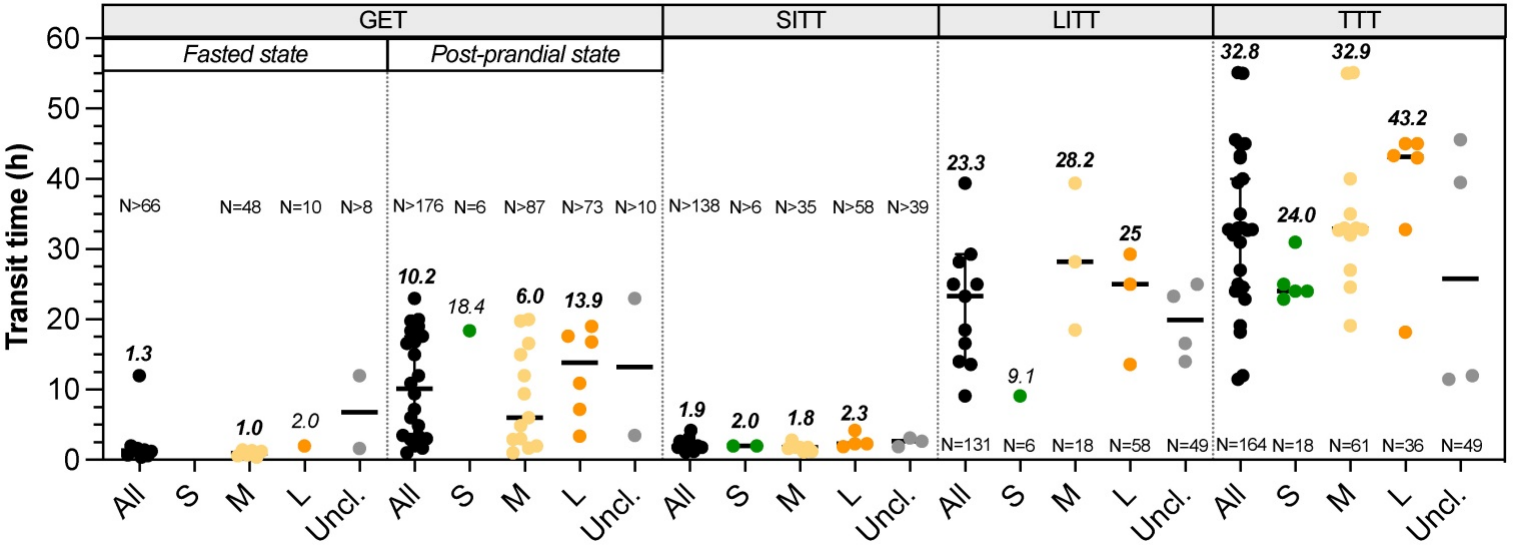
**Impact of dog sizes on gastrointestinal transit time.** Results from studies in dogs evaluating gastric emptying time (GET) under fasted or fed status, small intestinal transit time (SITT), large intestinal transit time (LITT) and total transit time (TTT) are represented. The same caption as used in **Fig. 1** was applied

**Transit time.** There is no available data on the duration of oral phase in dogs, but they are well known to quickly swallow their whole food. Data on gastric emptying time (GET), small intestinal transit time (SITT), orocaecal transit time (OCTT), large intestinal transit time (LITT) and total transit time (TTT) can be found in the literature with homogeneous definition between studies (**Fig. 4**, **Table S4**). Three different studies evaluate the impact of dog size on GET fed animals. Weber *et al.* 75 showed no significant difference in half-gastric emptying time between four breeds dogs (i.e. Miniature Poodle, Standard Schnauzer, Giant Schnauzer and Great Danes) using radiopaque markers ingested with food (T_50_ = 6.4-7.8 h). Without specifying any values, Bourreau *et al.* 89 concluded on a longer GET in large compared to small breeds after ingestion of a dry food meal using breath test method. Contrarily, Boillat *et al.* 4 described a shorter GET in large compared to medium breeds (range 6.8-15 h), using wireless motility capsule immediately administered after a dry food meal. Thus, there is apparently no relationship between BW and GET not only in fed, but also in fasted animals (**Fig. 4**). Besides, a liquid meal conduced to a shorter GET compared to meat, with 90% emptying in 0.4 h and 50% in 1-3 h, respectively (unknown dog size and method) 90. This suggests that canine gastric emptying is influenced by food consistency 91. There is also no consensus on the effect of dog size on SITT. Oswald *et al.* 43 and Weber *et al.* 8 found no influence of breed or BW, while Boillat *et al.* 4 measured a shorter SITT in largest dogs, ranging 1.6-3.7 h without linking transit time and dog size 4,8,43. OCTT was evaluated in dogs using very different methods. Some studies used sulfasalazine (converted into sulfapyridine in plasma) but do not employ the same threshold to define OCTT, i.e. either 50% conversion or first appearance in plasma 75,92, whereas more recent studies used wireless motility capsule. As a consequence, extremely variable results of OCTT are provided, from 2.2-2.8 h with sulfapyridine 75,92 to 20.7 h with capsule 93. Whatever the method used, these authors conclude to an absence of correlation between OCTT and BW. Studies of Boillat *et al.* 4 and Warrit *et al.* 94 assessed LITT in dogs from several breeds and various BW using wireless motility capsule. Both works conclude on the absence of correlation between LITT and BW (**Fig. 4**), with T_1/2_ ranged 7.1-42.9 h 4 and 25.0 h (1.1-49.1 h) 86. However, using plastic beads, researches revealed a longer LITT in large dogs (29.3 h for great Dane) than in small dogs (9.1 h for Miniature Poodle) and a significant positive correlation between LITT and BW, but also between LITT and shoulder height was demonstrated 92. In this study, LITT accounts for 39% of mean TTT for small breed dogs and 70% for large ones, which means that longer transit times observed in large dogs could be related to a longer LITT. Lastly, TTT showed a clear positive correlation with BW, as highlighted in **Fig. 4** 18. When gathering the data obtained in all the available studies, TTT ranged from 22.9-31 h (calculated median 24 h) in small dogs, 19.1-55 h (median 32.9 h) in medium dogs and 18.2-45 h (median 43.2 h) in large dogs. Especially, using plastic beads in small and large breeds, TTT observed was 22.9 h in Miniature Poodle and 43.3 h in Great Dane, whilst giant Schnauzer showed an even higher TTT of 55 h 95. This result was explained by the authors through a high stress sensitivity of giant Schnauzer that could influence their transit time in refraining their defecation, emphasizing a breed effect in addition to body size influence.

## Impact of body size on microbial parameters

### Gut microbiota composition

**Figure 5:**
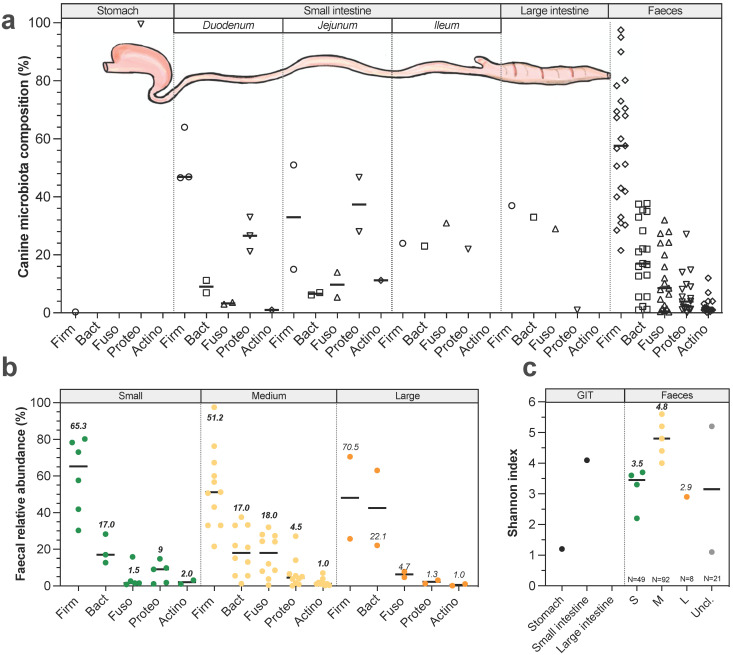
** Variations in gut microbiota composition along the canine digestive tract and impact of dog sizes.** Main bacteria populations found in the different compartments of the dog gastrointestinal tract are represented in (a). Bacteria counts are expressed in colony forming units (CFU) per gram of digestive content. Results from studies exploring by 16S rRNA Illumina sequencing canine microbiota composition (regardless of dog size) in the different digestive compartments are presented in (b). Influence of dog sizes on faecal microbiota composition at the phylum level is shown in (c) and corresponding Shannon index in (d). Canine main phyla are *Firmicutes* (Firm), *Bacteroidetes* (Bact), *Fusobacteria* (Fuso), *Proteobacteria* (Proteo) and *Actinobacteria* (Actino). The same caption as used in **Fig. 1** was applied

**Longitudinal variations.** In dogs like in other mammals, microorganisms colonize the entire GIT from mouth to rectum. All along GIT, there are longitudinal variations in gut microbiota composition due to changes in pH, substrate concentrations (including oxygen and nutrient availability) and transit time 64,96,97. Gut microbiota has been weakly described in dogs (compared to humans) and most of available studies have been performed since 2003 (for detailed information see **Table S5**). Canine oral microbiota present similar number (around 350 bacterial taxa from 148 genera) but significantly different populations compared to the human ones 98 and is mainly colonized by *Proteobacteria* (45%), *Bacteroidetes* (25%) and *Firmicutes* (19%). Most abundant species are *Porphyromonas cangingivalis* and *Porphyromonas gulae* 99,100. Regarding the other digestive compartments, studies have been mostly performed on faeces to avoid invasive procedures. Stomach is the less colonized compartment with 10^4^ to 10^5^ colony forming units (CFU) per gram of content in medium dogs, mainly composed by *Proteobacteria* (**Fig. 5A**) including *Helicobacter spp.* that are potential pathogenic strains 31,96,101. Small intestine contains 10^5^ to 10^7^ CFU/g of content 31,101. Duodenum (**Fig. 5A**) is colonized by *Firmicutes* (calculated median 47%), *Proteobacteria* (27%), *Bacteroidetes* (9%), *Fusobacteria* (3%) and *Actinobacteria* (1%), whereas jejunum is characterized by a higher abundance in *Proteobacteria* (37%), *Actinobacteria* (11%) and *Fusobacteria* (10%), together with lower percentages of *Firmicutes* (33%) and *Bacteroidetes* (7%) 28,102,103. Ileum (**Fig. 5A**) is dominated by 31% *Fusobacteria*, 24% *Firmicutes*, 23% *Bacteroidetes* and 22%* Proteobacteria* 104. These abundances should be considered with caution as they have been found in a single study performed in 6 medium dogs. As for other mammals, large intestine is the most colonized part of the GIT, with up to 10^9^ to 10^11^ CFU/g of content 96. According to a unique publication using 16S Illumina sequencing to investigate microbiota composition from 6 healthy Hound dogs 104, colonic digesta is dominated by 37%* Firmicutes*, 33% *Bacteroidetes*, 29% *Fusobacteria* and 1% *Proteobacteria* including *E. coli*-like organisms (**Fig. 5A**). It's interesting to highlight that majority of taxa colonizing the colon are also found in canine faeces 105 which seems to be rather different from the human situation where a significant number of mucus-adherent bacteria from the colon are not found in faeces 105. No study has investigated dog size effect on gut microbiota composition elsewhere than in stools, and the main variations in faeces are presented in **Figure 5B** and** 5C**. Whatever dog sizes, faecal microbiota of healthy dogs is dominated by three main bacterial phyla: *Firmicutes*,* Bacteroidetes* and *Fusobacteria* 105. Bacteria from *Actinobacteria* and *Proteobacteria* phyla are also found in canine faeces but in a lesser proportion. Of interest, a variable relative abundance of *Bacteroidetes* was reported and was inversely correlated to *Fusobacteria* relative abundance indicating they might occupy the same ecological niches 106. *Fusobacteria* and *Proteobacteria* seem to be more abundant in dogs than in other omnivorous, probably related to diet changes 107. Unlike in human where *Fusobacterium* is frequently associated with diseases, in dogs this genus is related to non-stressful conditions and is therefore probably a marker of an healthy state, especially because its abundance increases when dogs have access to the outside 43. In small dogs faeces (**Fig. 5B**), average *Firmicutes* proportions vary widely from 30 to 80%, followed by 13-28% *Bacteroidetes*, while a lower abundance of *Proteobacteria* (1-15%), *Fusobacteria* (1-16%) and *Actinobacteria* (1-3%) was detected 108-110. Medium dogs display similar value ranges of *Firmicutes* (15-98%), *Bacteroidetes* (0.1-34%), *Proteobacteria* (0.1-27%) and *Actinobacteria* (1%), but a larger proportion of *Fusobacteria* (0.1-40%) compared to small dogs 108,111. Only one study investigated faecal microbiota composition in 8 large dogs and quantified 71% *Firmicutes*, 22% *Bacteroidetes*, 5% *Fusobacteria,* and 1% *Actinobacteria*, with interestingly a much lower abundance of* Proteobacteria* (1%) than in small and medium dogs 40. In few studies, canine faecal diversity was followed with Shannon index and calculated medians seem to be higher in medium dogs (4.8, four studies) compared to small (3.5, five studies) and large dogs (2.9, a single study) (**Fig. 5C**). In addition to Bacteria (representing 98%), canine faecal microbiota also contains 1.1% Archaea, 0.4% Fungi and 0.4% viruses, mainly bacteriophages 112,113. Fungal part of the faecal microbiota is composed by 97.9% *Ascomycota* and 1% *Basidiomycota* 114. Even if methanogen Archaea have been detected in healthy dogs faeces, there is no information on their methanogen potential 114.

**Radial variations.** In addition to longitudinal variations, there are also radial changes in gut microbial composition that starts to be described in human 115 but are still in infancy in dogs. Indeed, the entire gut epithelium is covered by a mucus layer that offers an alternative source of host-derived nutrients. This mucus is colonized by a specific mucus-adherent microbiota (namely mucosal microbiota) and seems to play a key role in host homeostasis 116. Of note, there is a lack of studies on the canine mucosal microbiota. Only two studies investigated the mucosa-associated bacteria on the outer mucus layer in the colon of healthy dogs, using targeted FISH approach 117,118. Analysis of colonic biopsy samples from healthy Boxers revealed that bacteria appear to be restricted to the outer mucus layer, as no bacteria was detected within the mucosa 117. In addition, Cassmann *et al.* 118 demonstrated that free ileal and colonic mucus of healthy young dogs (< 2 years old) was mainly colonized by *Bacteroidetes spp*. and *Eubacteria,* while *Eubacteria* represented the major bacteria attached to adherent mucus. Authors reported that there were almost no bacteria attached to surface epithelium or contained within mucosa. Of interest, *Akkermansia muciniphila*, a well-known mucin-degrading bacteria in humans, inversely correlated to obesity, was not yet identified in canine faeces 119.

### Gut microbiota metabolic activities and functions

**Figure 6:**
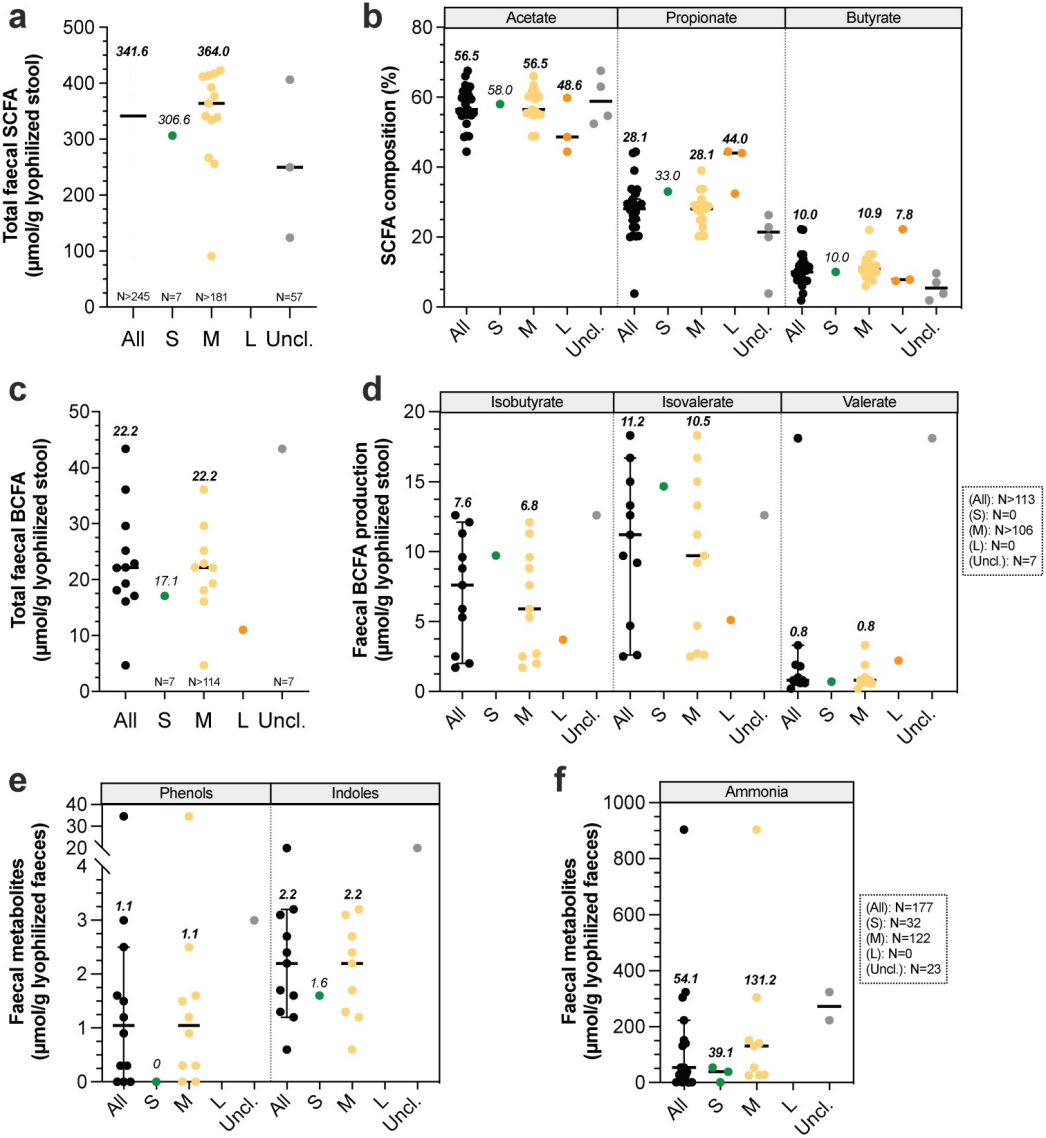
**Impact of dog sizes on faecal microbial products production.** Results from studies in dogs measuring total faecal major short-chain fatty acids (SCFA, i.e. acetate, propionate and butyrate) are presented in (a) and detailed in (b). Similarly, influence of dog size on major branched-chain fatty acids production (BCFA, i.e. isobutyrate, isovalerate and valerate) is shown in (c) and detailed in (d). Effect of dog size on other microbial metabolites are presented in (e) for phenols and indoles and (f) for ammonia. The same caption as used in **Fig. 1** was applied

Gut microbiota is known to play a key role in host homeostasis and health maintenance, as it is implicated in many nutritional (e.g. vitamin synthesis, fibre degradation), immunological (immune system maturation) and physiological processes (e.g. vascularization, epithelium integrity, “barrier” effect against pathogens and lipid digestion via the metabolism of primary BA into secondary BA) 20,120. At a functional level, whatever the type of food, identified gene content of microbiome from medium dogs was not modified and was associated with the metabolism of carbohydrates (12.5-13%), proteins (8.1-9.1%), DNA (7.1-7.4%), cell wall and capsule (7-7.6%), amino acids and derivatives (6.8-6.9%), cofactors, vitamins, prosthetic groups and pigments (5.7-6%) and bacterial virulence (6.2-7.2%) 112. These results underline that all microbiota functions are far to be already discovered, as proved by the remaining 42.8% non-affiliated genes. Guard and Suchodolski 121 have studied faeces from 8 healthy dogs (2.7 to 31.8 kg) and observed high inter-variability microbiota composition between animals, while bacteria's functions were very consistent. Thus, even if gut microbiota composition highly vary between dogs, the functional potential seems to be unchanged whatever dog sizes 20. Gut microbiota metabolic activity leads to gas and short-chain fatty acid (SCFA) production from soluble fibres. SCFAs stimulate intestinal motility and can be further used as an energy source for colonocytes, liver and brain. The three main SCFAs are acetate, propionate and butyrate, with faecal relative percentages of 60:25:15 122. Non-digested protein from diet and endogen proteins are also metabolized by gut microbiota, leading to the production of branched chain fatty-acids (BCFA), ammonia, indoles and phenols 18. Canine faecal protein degradation products are associated with deleterious effects, such as poor faecal quality, inflammation and kidney diseases in dogs and colorectal cancer in humans 123,124. Canine SCFA production was only evaluated in faecal samples (**Fig. 6A-B, Table S6**). Values were mainly obtained in medium dogs (especially Beagles) and are widely variable due to many differences in design study (e.g. type of food, food composition in carbohydrates, methods, type of units). However, in a study performed by Weber *et al.* 36 , the authors compared SCFA production between small, medium and large dogs and demonstrated that total SCFA concentration in stool significantly increased with BW, with 448 ± 67, 894 ± 80 and 1184 ± 259 mmol/kg of lyophilized faeces for small, medium and large dogs respectively. This is consistent with a longer LITT in large breed dogs that may promote microbial fermentation. Large quantity of organic acids produced could thus exceeds colonic mucosa absorption capacity, thereby leading to an accumulation in lumen, a decrease of colonic pH and an increased faecal excretion 18. Similarly, total BCFAs were measured only in faecal samples, and mainly in medium dogs (**Fig. 6C**, **Table S6)**. BCFA concentrations seem to be lower in small dogs (a unique value of 17.1 µmol/g) compared to medium ones (calculated median of 22.2 µmol/g). Moreover, BCFA composition was only studied in medium dogs, with a calculated median concentration of 6.8 µmol/g isobutyrate, 10.5 µmol/g isovalerate and 0.8 µmol/g valerate (**Fig. 6D**). Phenols, indoles and ammonia concentrations were also poorly studied in small and medium dogs and to our knowledge never measured in large dogs (**Fig. 6E-F**). Based on our calculated medians, it appears that these products are found in higher concentrations in medium than in small dogs. Lastly, to our knowledge there is no data on gas production in dogs and the two studies on gas composition focused on malodorous compounds such as hydrogen sulphide 125,126.

## Discussion and general conclusion

**Figure 7:**
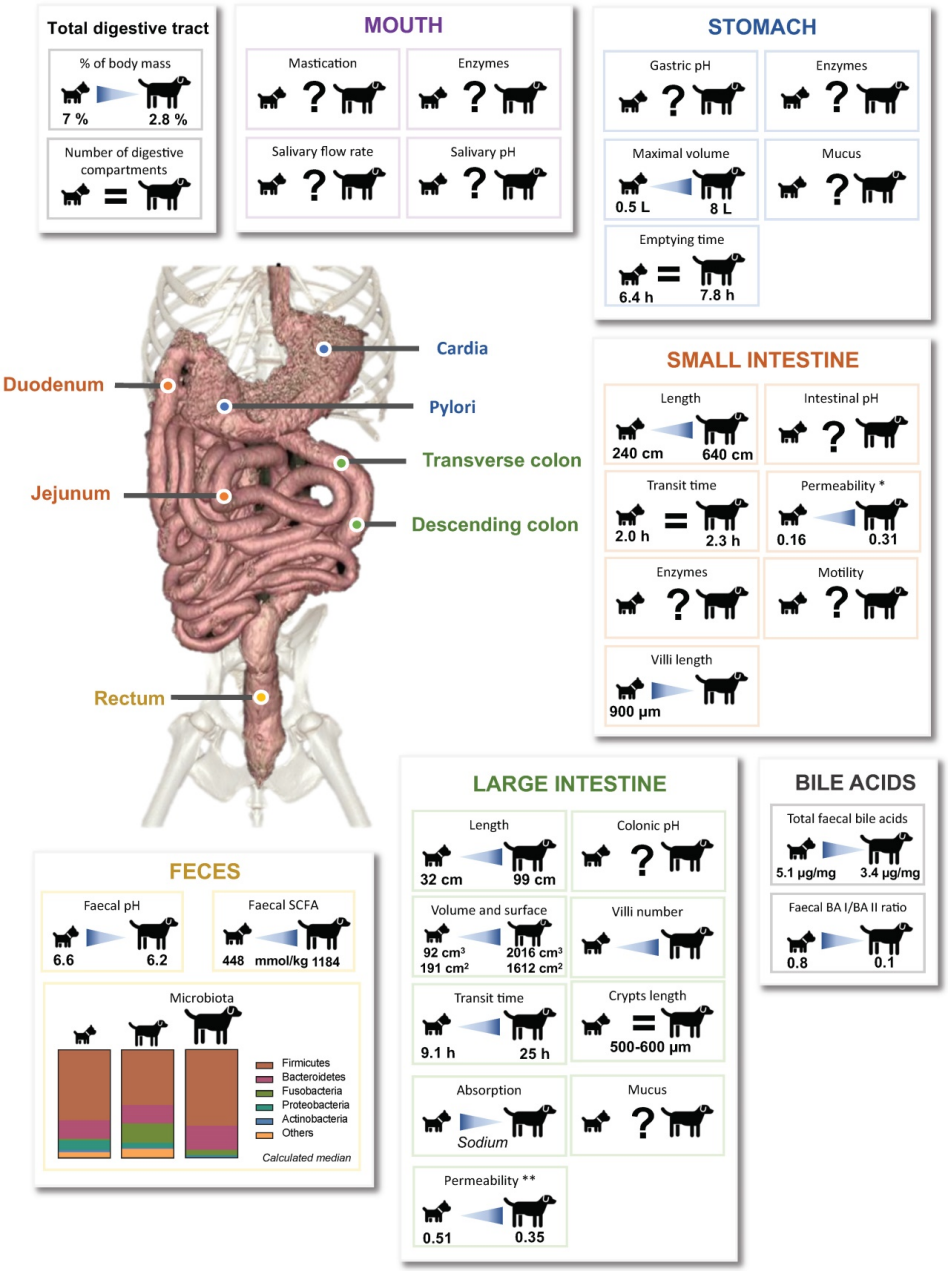
Overview of the impact of dog sizes on digestive physiology and faecal microbiota composition and activity. Key parameters of the oral, gastric, intestinal and colonic compartments from the canine digestive tract are summarized. Specified values were obtained from reports comparing in a same study the results obtained for small and large dogs. Lack of data are represented by “?”, BA: bile acid, SCFA: short chain fatty acids. *: Lactulose/L-rhamnose ratio, **: Lactulose/sucralose ratio

In an original way, this review gives an overview of available literature concerning the effect of dog sizes (i.e. “small”, “medium” and “large” sizes) on digestive anatomy and associated physicochemical and microbial parameters, illustrating data with both synthetic graphs (**Fig. 1 to Fig. 6**) and exhaustive tabs (**Table S1 to S6**). Even if our conclusions may be hampered by the paucity and old age of many data, as well as the huge variability between experimental protocols (diet composition, measurement methods and data analysis processes) and animals (live or dead, anesthetized or not, companion or laboratory animals, environment), we evidenced clear effects of dog's BW on gastrointestinal physiology, mainly in relation with the colonic compartment (**Fig. 7**). Large intestine length, area and volume clearly increase with dog size. This seems to be associated with a higher colonic transit time that can affect nutrient and water absorption, gut microbiota composition and activity, as well as faecal moisture. Thus, sodium and potassium absorption are lower in larger dogs resulting in a higher concentration in faecal samples. Large dogs are also characterized by a higher intestinal permeability that can induce a backflow of absorbed electrolytes into the colonic lumen, translated into a luminal retention of electrolytes and water 18. Besides, a longer colonic residence time in large dogs should promote microbial fermentations and especially a higher fibre degradation by resident bacteria. This higher fermentation capacity results in a stronger production of SCFAs leading to a diminution in faecal pH, and to a potential disturbance of water absorption due to the high osmotic power of SCFAs 36. Together with an increased colonic permeability, excessive SCFAs production would induce water retention in the colon, associated with higher faecal water content and loose watery stools frequently observed in large dogs 43,127. In addition, faecal concentrations of microbial degradation products from proteins (phenol, indole, ammonium and BCFAs) seem to be positively associated with dog BW, which again may be explained by a longer transit time. Moreover, our data analysis suggests an increase in *Fusobacteria* according to BW (observed between small and medium dogs), which can be related to an increase in protein metabolites 65,124. As certain bacteria are fully involved in BA deconjugation, changes in microbiota composition depending on dog's BW can also be linked to modifications in BA concentrations, inversely correlated with BW.

Further studies would be necessary to enhance available data on physicochemical parameters, especially in the upper GIT, but also on gut microbiota that remains very poorly described in each digestive compartment and not described at all in the mucus layer. Lastly, our bibliographic review revealed the large predominance of some breeds (i.e. Miniature Poodle, Beagle, Standard and Giant Schnauzer and Great Dane) and breeds showing well-known specific digestive particularities (like German Shepherd) or specific energy needs (like Husky, Great Danes or Terriers) 80. It would be therefore of high interest to further analyze current data by considering not only the effect of body size but also that of breeds. Taken together, all the specificities raised in large dog digestive physiology may be correlated to their high sensitivity to diet and digestive diseases 18. Finally, all these data concerning the effect of dog size on their digestive physiology can be helpful for the development of new food or veterinary products at the individual level, in accordance with a personalization step intended by petfood and pharma companies. In full accordance with the 3R rules (aiming to reduce animal experiments), such *in vivo* data also provide key information necessary to develop and validate *in vitro* gut models adapted to each dog sizes for in-depth mechanistic studies on dog digestive physiology 128.

## Supplementary Material

Supplementary tables.

## Author contributions

SBD, EA and DH had the idea to make a literature review on this topic and designed the review. CD performed the literature survey, data analysis and figures design. SBD and CD wrote the first draft of the manuscript. All authors critically revised and approved the manuscript.

## Abbreviations

BA: bile acids
BCFA: branched-chain fatty acids
BW: body weight
CA: cholic acid
CDCA: chenodeoxycholic acid
CFU: colony forming units
DCA: deoxycholic acid
GET: gastric emptying time
HCl: hydrochloric acid
LCA: lithocholic acid
LITT: large intestine transit time
OCTT: orocaecal transit time
SCFA: short-chain fatty acids
SITT: small intestine transit time
TTT: total transit time
