# Supplementary material for: From Chihuahua to Saint-Bernard: how did digestion and microbiota evolve with dog sizes
Source: Int J Biol Sci. 2022 Aug 1;18(13):5086–102. doi: 10.7150/ijbs.72770 (PMC9379419; doi:10.7150/ijbs.72770)
Supplement: Supplementary file 1 — Supplementary tables. [file ijbsv18p5086s1.pdf]

<u>Supplementary Table S1</u>: Overview of studies reporting pH of healthy adult dogs depending on body weight in the different compartments of the digestive tract.

References are organized according to dog sizes (small, medium, large, unclassified) in reverse chronological order. FOS: fructooligosaccharide; HP: high protein; IF: insoluble fiber; LP: low protein; N/A: not available; SF: soluble fiber; TDF: total dietary fiber.

References	N=	Weight (kg)	Age (years)	Breed	Food				Methods	Stomach		Small intestine	Large intestine	Faeces
					Type of food	Protein (%)	Fat (%)	Fiber (%)		Fasted	Fed			
**SMALL**														
Igarashi *et al.* 2017	25	6.1	8.6 (2-16.4)	N/A	N/A				pH probe	N/A	N/A	N/A	N/A	6.4 (5.5-7.4)
Beloshapka *et al.* 2014	12	8.4 ± 1.2	3.3 (1-6)	Miniature Schnauzer	Balanced canned diet	36.1	25.4	8.9<br>SF: 4.5<br>IF: 4.4	pH probe	N/A	N/A	N/A	N/A	6.8
Weber *et al.* 2004	6	3.5	1.25	Miniature Poodle	Experimental dry food (Royal Canin)	39.2	16.3	9.8	pH probe	N/A	N/A	N/A	N/A	6.5
**MEDIUM**														
Alexander *et al.* 2019	12	10.4 ± 0.9	3.2 ± 0.8	Beagles	Experimental diet	38.6	17.3	7.2	pH probe	N/A	N/A	N/A	N/A	6.6
Detweiler *et al.* 2019	8	12.8 ± 1.7	4.6 ± 0.6	Beagles	Experimental diet	31.7	11.9	SF: 2.6<br>IF: 2.4	pH probe	N/A	N/A	N/A	N/A	6.3
Eisenhauer *et al.* 2019	10	13.7	4.4 ± 0.3	Beagles	Experimental dry diet	21.9	9.8	TDF: 3.4<br>Crude fiber: 0.9<br>IF: 1.9<br>SF: 1.5	pH probe	N/A	N/A	N/A	N/A	6.9
Koziolek *et al.* 2019	6	13-18	2-4	Beagles	Dry or canned commercial food	N/A			Telemetric motility capsule (SmartPill®)	N/A	N/A	Proximal: 6.5-7<br>Distal: 7.5-8	5-8	N/A
Nogueira *et al.*, 2019	8	18 ± 1.4	4.2 ± 1.1	N/A	Experimental diet	30.8	18.5	12.0<br>SF: 3.2<br>IF: 8.9	pH probe	N/A	N/A	N/A	N/A	6.0
Algya *et al.*, 2018	8	13	3.6 ± 0.3	Beagles	Commercial dry diet	24.1	13.3	9.6	pH probe	N/A	N/A	N/A	N/A	6.2 ± 0.2
Pinna *et al.*, 2018	N/A	19.5 ± 6.2	3.6 ± 1.6	N/A	LP diet, LP diet + FOS, HP diet, HP diet + FOS	N/A			pH probe	N/A	N/A	N/A	N/A	LP: 6.1<br>LP+FOS: 6.5<br>HP: 6.6<br>HP+FOS: 6.5
Herstad *et al.*, 2017	11	19.7 (10.3-28.7)	4.8	English springer spaniel, 3 mixed breads, Small Munster lander, Eurasier, 2 Irish setter, 2 English cocker spaniel, German shorthaired pointer	Commercial dry food	27.1	16.3	1.2	pH probe	N/A	N/A	N/A	N/A	6.5 (6.2-7.1)
Beloshapka *et al.*, 2016	48	10	3.4	Beagles	Experimental diet	30.1	18.6	5.7<br>SF:1.5<br>IF: 4.2	pH probe	N/A	N/A	N/A	N/A	6.5
Wambacq *et al.*, 2016	8	11.6	5-5.5	Beagles	Experimental diet supplemented with SF and IF	16.6	14.6	1.8<br>SF: 2<br>IF: 4.7	pH probe	N/A	N/A	N/A	N/A	SF: 5.7<br>IF: 6.5
Hang *et al.*, 2013	5	19 (18.3-19.3)	5	Beagles	Commercial diet	N/A			pH probe	N/A	N/A	N/A	N/A	6.9 (6.8-7.1)

References	N=	Weight (kg)	Age (years)	Breed	Food				Methods	Stomach		Small intestine	Large intestine	Faeces
					Type of food	Protein (%)	Fat (%)	Fiber (%)		Fasted	Fed			
Beloshapka, Wolff, & Swanson, 2012	8	20 ± 0.5	3.5 ± 0.5	Mixed breed	Experimental diet	35.2	20.2	8.6	pH probe	N/A	N/A	N/A	N/A	6.7
Garcia-Mazcorro *et al.*, 2012	8	18.6 ± 2.0	0.8	Mixed breed	Commercial diet	N/A	N/A	4%	pH probe	1.7 (1.5-1.9)<br>15 days later:<br>1.8 (1.5-2.1)	N/A	N/A	N/A	N/A
Mahar *et al.*, 2012	14	8-14	1-6	Beagles	N/A	25.5	18	2.8	Bravo pH telemetry system. attached to the gastric mucosa	1.5	0-1 h: 1.6<br>1-2h: 1.7<br>2-6h: 1.5	N/A	N/A	N/A
Faber *et al.*, 2011	4	22 ± 2.1	3.4	N/A	Experimental dry diet	31.6	21.6	11.5	pH probe	N/A	N/A	N/A	N/A	6.7
Mentula *et al.*, 2005	22	12-19	1-3	Beagles	Canned dog food	N/A			pH probe	N/A	N/A	Jejunum:<br>6.8 (6.4-7.5)	N/A	6.6<br>(6.1-7.4)
Weber *et al.*, 2004	6	12.6 ± 0.9	1.25	Standard Schnauzer	Experimental dry food	39.2	16.3	9.8	pH probe	N/A	N/A	N/A	N/A	6.6
	6	23.3 ± 1.3	1.25	Giant Schnauzer						N/A	N/A	N/A	N/A	6.4
Zentek *et al.*, 2004	8	12.4 ± 2.2	1-11	Beagles	Commercial diet	31.0	14.6	2.5	pH probe	N/A	N/A	N/A	N/A	6.5
Flickinger *et al.*, 2003	4	12 ± 1.3	3	Beagles	Commercial diet	N/A			pH probe	N/A	N/A	N/A	N/A	5.6<br>6.1
Yago *et al.*, 1997	6	15-20	N/A	Mixed breeds	Commercial diet with sunflower (S) or olive oil (O)	N/A	9 or 15	N/A	N/A	N/A	N/A	Duodenum:<br>9% lipids:<br>S: 5.7<br>O: 4.7<br><br>15% lipids:<br>S: 6.8<br>O: 6.4	N/A	N/A
Shinchi *et al.*, 1996	8	10-15	N/A	Mixed breed	N/A				pH probe	N/A	3.2 ± 0.3	Duodenum: 6.6 ± 0.2	N/A	N/A
Gupta & Robinson, 1988	3	15-20	"adult"	Mixed breed	25, 50-, 100-, 300- and 500-mL water	N/A			N/A	N/A	N/A	Duodenum: 7.7<br><br>25: 6.9-7.8<br>50: 6.5-7.8<br>100: 4.8-7.6<br>300: 2.9-7.2<br>500: 2.1-8.0	N/A	N/A
Lui *et al.*, 1986	4	10-20	2-3	Beagles	Purina laboratory canine diet	N/A			Heidelberg capsule	1.8<br>± 0.1	N/A	N/A	N/A	N/A
Ehrlein & Pröve, 1982	5	13-17	N/A	Beagles	Low, medium, and high viscosity diet	N/A			N/A	N/A	N/A	Mean duodenal output: 7<br>30 min after eating: 5	N/A	N/A
Banta *et al.*, 1979	12	N/A	"adult"	Beagles	Meat diet	N/A			N/A	N/A	5.9<br>8h after eating: 2.4	Proximal duodenum:<br>6.2 (6-7.2)	N/A	N/A

References	N=	Weight (kg)	Age (years)	Breed	Food				Methods	Stomach		Small intestine	Large intestine	Faeces
					Type of food	Protein (%)	Fat (%)	Fiber (%)		Fasted	Fed			
Smith, 1965	3	N/A	“adult”	Beagles	N/A				pH probe	N/A	Anterior: 5.5<br>Posterior: 3.4	Beginning: 6.2<br>End: 7.5<br>Cecum: 6.4	6.5	6.2
**LARGE**														
Sandri *et al.*, 2016	8	30.3 ± 3	4.2 ± 2.8	Boxer	Commercial dry food	26.7	10.6	2.8	pH probe	N/A	N/A	N/A	N/A	6.5 ± 0.2
Cutrignelli *et al.*, 2009	4	GS: 32.5<br>MN: 59.8	3	2 German Shepperd<br>2 Mastiff Neapolitans	Commercial dry food	25.8	N/A	6.6	pH probe	N/A	N/A	N/A	N/A	5.6
Kalantzi *et al.*, 2006	4	29-35	N/A	Labrador	250 mL water	N/A			N/A	N/A	N/A	Jejunum:<br>Fasted: 7.1<br>Fed: 6.0	N/A	N/A
Weber *et al.*, 2004	6	46.3 ± 1	1.25	Great Danes	Experimental dry food	39.2	16.3	9.8	pH probe	N/A	N/A	N/A	N/A	6.5
**UNCLASSIFIED**														
Duysburgh *et al.*, 2020	N/A	N/A	N/A	N/A	N/A				pH probe	N/A	2.0	6.8	6.7-6.9	N/A
Warrit *et al.*, 2017a	42	31.4 (11.8-63.8)	4 (1-11)	Mixed breed	Dry food (Purina)	N/A			Wireless motility capsule	1.7 (1-2.3)	1.8 (0.8-2.7)	7.8<br>(7-8.9)	6.4<br>(5.3-8.2)	N/A
Warrit *et al.*, 2017b	42	31.4 (11.8-63.8)	4 (1-11)	Mixed breed	Dry food (Purina)	N/A			Wireless motility capsule	1.7 (1.5-1.9)	N/A	7.9<br>(7.3-8.8)	6.2<br>(5.8-7.4)	N/A
Lidbury *et al.*, 2012	8	29.1 (22.1-41)	1.75 (0.8-11)	5 mixed breeds, Flat-coated Retriever, German Shepherd, Golden Retriever	N/A				Wireless motility capsule	1.6 (0.9-2.7)	N/A	7.6<br>(7.3-8.2)	5.7<br>(4.9-7.3)	N/A
Sagawa *et al.*, 2009	16	12 (9.5-14.5)	4.7 (3-6)	Beagles	10 or 200 g of meal	N/A			Bravo pH telemetry system	2.0 ± 0.6	10 g: 1.1<br>200 g: 1.3	7-7.5	N/A	N/A
Biagi, Cipollini, & Zaghini, 2008	8	N/A	N/A	N/A	N/A				pH probe	N/A	N/A	N/A	N/A	6.5
Martinez, 2002	N/A	N/A	N/A	N/A	N/A				N/A	N/A	Posterior: 3.5	Duodenum: 6.1<br>Jejunum: 6.6<br>Ileum: 7.5	6.4	N/A
Kararli, 1995	N/A	N/A	N/A	N/A	N/A				N/A	1.5	2.1 ± 0.1	N/A	N/A	N/A
Stevens & Hume, 1995	N/A	N/A	N/A	N/A	N/A				N/A	N/A	N/A	6.0-7.4	6.0-7.3	N/A
Dressman, 1986	N/A	N/A	N/A	N/A	N/A				N/A	1.5 (0.9-2.5)	2.1<br>(0.5-5)	« 1 unit more than in human »	N/A	N/A
Lui *et al.*, 1986	N/A	N/A	N/A	N/A	N/A				N/A	N/A	N/A	20min after gastric emptying:<br>7.7<br>180min after:<br>7.2	N/A	N/A
Gruber *et al.*, 1987	N/A	N/A	N/A	N/A	N/A				N/A	N/A	N/A	Duodenal effluents:<br>before particles discharge: 6.1 (2.4-7.6); after: 7.4 (5.7-8.3)	N/A	N/A
Florey & Harding, 1934	N/A	N/A	N/A	N/A	N/A				N/A	N/A	N/A	Duodenum: 8.4	N/A	N/A

References	N=	Weight (kg)	Age (years)	Breed	Food				Methods	Stomach		Small intestine	Large intestine	Faeces
					Type of food	Protein (%)	Fat (%)	Fiber (%)		Fasted	Fed			
												Duodenal content: 8.7-8.9		

<u>Supplementary Table S2</u>: Overview of studies reporting saliva, gastric juice and pancreatic juice composition in healthy adult dogs depending on body weight.

References are organized according to dog sizes (small, medium, large, unclassified) in reverse chronological order. BA I: primary bile acid; BA II: secondary bile acid; BA: bile acids; Bic: bicarbonate (NaHCO3); BW: body weight; Ca: Calcium; CA: cholic acid; CDCA: chenodeoxycholic acid; Cl: chloride; CO2: carbon dioxide; DCA: deoxycholic acid; GC-MS: gas chromatography mass spectrometry; GC-TOF-MS: gas chromatography time-of-flight mass spectrometry; GC: gas chromatography; GCA: glycocholic acid; GLC: gas liquid chromatography; GLCA: glycolithocholic acid; GUDCA: glycoursodeoxycholic acid; HPLC: high performance liquid chromatography; K: potassium; LC-MS/MS: liquid chromatography coupled to tandem mass spectrometry; LCA: lithocholic acid; LDH: lactate dehydrogenase; Mg: magnesium; N/A: not applicable; Na: sodium; NA2CO3: sodium carbonate; P: phosphor; SDS-PAGE: sodium dodecylsulfate polyacrylamide gel electrophoresis; TCA: taurocholic acid; TCDCA: taurochenodeoxycholic acid; TDCA: taurodeoxycholic acid; TLCA: taurolithocholic acid; TUDCA: tauroursodeoxycholic acid; UDCA: ursodeoxycholic acid; Zn: zinc.

References	N=	Weight (kg)	Age (years)	Breed	Type of food	Methods	Saliva	Gastric juice	Pancreatic juice	Bile	
										Place of measure	Composition
**SMALL**											
Guard *et al.*, 2019	4	5.0	4.0	Fox Terrier, Schnauzer/Yorkie, Dachshund, Mixed Breed, Jack Russell Terrier	N/A	N/A	N/A	N/A	N/A	Faeces (µg/mg lyophilized)	CA: 0.4<br>CDCA: 0.2<br>LCA: 0.5<br>DCA: 1.7<br>UDCA: 0.03<br>BA I: 0.6<br>BA II: 2.2<br>Total: 2.8
**MEDIUM**											
Guard *et al.*, 2019	17	23.3	5.0	2 Boston Terrier, Boxer, Mixed Breed, 2 Labrador Retriever, Aussie, Labradoodle, Belgian Malinois, English Setter, Chocolate Lab, Mixed Breed, Golden Retriever, English Pointer	N/A	N/A	N/A	N/A	N/A	Faeces (µg/mg lyophilized)	CA: 0.5<br>CDCA: 0.3<br>LCA: 1.1<br>DCA: 2.8<br>UDCA: 0.03<br>BA I: 0.7<br>BA II: 3.9<br>Total: 4.7
Hong *et al.*, 2019	16	N/A	N/A	Beagles	N/A	Commercial enzyme kit	Amylase: 37.3 (15.4-52.7) UI/L	N/A	N/A	N/A	N/A
Tecles *et al.*, 2018	18	24.2 ± 11.7	2 (1.8-5)	7 Mixed Breeds, 6 German Shepherd, 2 Labrador Retrievers, Dachshund, Bull Terrier, Jack Russell	N/A	Adenosine deaminase was measured using spectrophotometric automated method. Salivary amylase using a commercially available method.	Adenosine deaminase: 0.8 (0.1-3.2) IU/L or 0.4 (0-1.1) IU/g<br>Amylase: 26.5 (17.9-49.4) UI/L or 14.7 (6.6-21.3) UI/g	N/A	N/A	N/A	N/A
Kakimoto *et al.*, 2017	14	N/A	4 (3-8)	Beagles	N/A	HPLC	N/A	N/A	N/A	Gallbladder (mmol/L)	GUDCA: 0 (0-0.2)<br>TUDCA: 0.4 (0.1-0.8)<br>UDCA: 0.1 (0-0.2)<br>GCA: 1.6 (0-2.6)<br>TCA: 74.1 (55.7-154)<br>CA: 0.3 (0-3)<br>TCDCA: 16.8 (7.8-20.5)<br>TDCA: 31.1 (18-73.7)<br>TLCA: 0.6 (0-1.5)<br>Total: 125.8 (85.5-229.7)

References	N=	Weight (kg)	Age (years)	Breed	Type of food	Methods	Saliva	Gastric juice	Pancreatic juice	Bile	
										Place of measure	Composition
Yago *et al.*, 1997	6	15-20	N/A	Mixed breeds	Commercial diet with 9 % or 15 % lipids (sunflower)	Flow rates were determined gravimetrically	N/A	N/A	Flow rate (µL/min):<br>9%: 143.9 ± 20.3<br>15%: 127.5 ± 26.8<br>Bic (mEq/L):<br>9%: 65.7 ± 3<br>15%: 62.6 ± 5.1<br>Bic output (µEq/min):<br>9%: 11.1 ± 2.2<br>15%: 11.4 ± 3	N/A	N/A
Carrière *et al.*, 1992	6	14.5 (13.5-17.5)	2	Beagles	N/A	Stimulation of gastric juice secretion (urecholin). Collection every 15 min using a cannula<br><br>Gel filtration chromatography	N/A	Basal secretion:<br>Proton output: 0.4 mmol/15 min<br><br>Lipase:<br>190 ± 23 U/h	N/A	N/A	N/A
Yeo *et al.*, 1990	N/A	15-20	N/A	Mixed breeds	Fasted (smelling a meal)	Gastric juice was collected at low constant suction and measured at 15 min intervals	N/A	Peak gastric juice output: 10.8 ± 2.1 mEq/h	N/A	N/A	N/A
Wildgrube *et al.*, 1986	6	23.3 ± 1.3	1.25	Giant schnauzer	N/A	Jejunal canula and addition of secretin and cholecystokinin during 2 h.<br>Chromatography	N/A	N/A	N/A	Bile	TCA: 74.3 ± 4.6%<br>TDCA: 14.9 ± 3.8%<br>TUDCA: 5.3 ± 2.1%<br>Total BA:<br>With secretin:<br>74.6 µmol/mL<br>with cholecystokinin:<br>69.4 µmol/mL
Madrid *et al.*, 1983	36	20-25	"adult"	N/A	N/A	Total bilirubin from plasma was measured by oxidation with sulfanilic acid. TCA was estimated using Irvin *et al.* (1944) method. Chloride was estimated using potentiometric titration.	N/A	N/A	N/A	N/A	Basal secretion of bile:<br>29 mL/kg/24h<br>Bilirubin:<br>195 ± 14 mg/100 mL<br>Chloride: 70 ± 4.5 mmol/L<br>TCA: 70.5 ± 9 mg/mL

References	N=	Weight (kg)	Age (years)	Breed	Type of food	Methods	Saliva	Gastric juice	Pancreatic juice	Bile	
										Place of measure	Composition
Roblès Diaz *et al.*, 1981	12	13-25	N/A	7 Mixed Breeds,5 Beagles	Semisolid mixture (9% protein, 6% fat, 2% minerals, 70% water and 0.3% cellulose)	Pancreatic secretion was collected for 160 min in basal conditions and for 120 min after a meal of 450 g.<br><br>Bicarbonate concentration was determined by back-titration	N/A	N/A	Basal volume: 0.1 mL/kg BW/20 min<br>Postprandial peak secretion: 0.4 mL/kg/20 min<br>Total secretion: 1.5 mL/kg/120 min<br>Bic: Basal output: 3.5 μequiv/kg/20 min<br>Peak secretion: 44.5<br>Total secretion: 176.6/120 min<br>Concentration: 64.6 meq/L<br>Proteins:<br>Basal output: 1.6 mg/kg/20min<br>Peak secretion: 9.5 mg/kg<br>Total: 37.6 mg/kg/120 min<br>Concentration: 30.4 mg/mL<br>Trypsinogen:<br>basal output: 0.4 mg/kg/20min<br>Peak secretion: 1.8 mg/kg<br>Total: 7.4 mg/kg/120 min<br>Specific activity: 45.8 U	N/A	N/A
Ehrlein & Pröve, 1982	5	13-17	N/A	Beagles	3 types of meal: low (L). medium (M) and high (H) viscosity	Estimation of the volume of gastric secretion using a formula (Hunt, 1994).	N/A	Rate of emptying:<br>L: 1.4 ± 0.7 mL/min<br>M: 1.9 ± 0.4 mL/min<br>H: 2.0 ± 0.4 mL/min<br><br>Volume output:<br>L: 37.2 ± 23.3 mL<br>M: 119.8 ± 35 mL<br>H: 189.4 ± 49.6 mL	N/A	N/A	N/A
Beaver, Wostmann, & Madsen, 1978	N/A	N/A	0.4-0.5	Beagles	N/A	Samples of gallbladder are obtained from autopsy.<br>GLC	N/A	N/A	N/A	Gallbladder	CA: 81.6%<br>CDCA: 3.7%<br>DCA: 12.1%

References	N=	Weight (kg)	Age (years)	Breed	Type of food	Methods	Saliva	Gastric juice	Pancreatic juice	Bile	
										Place of measure	Composition
Henriksen & Worning, 1969	4	19-20	0.9-3.9	Mixed breed	Meat	CO_2 total concentration was determined according to Van Slyke's manometric method and is referred to as "bicarbonate". Protein concentration was estimated spectrophotometrically at 280 nm.	N/A	N/A	Mean volume (mL/min):<br>0-30 min: 0.5<br>30-60: 0.8<br>60-120: 0.8<br>120-180: 0.8<br>Mean Bic output (μequiv/min):<br>0-30: 59<br>30-60: 99<br>60-120: 98<br>120-180: 94<br>Mean proteins output (mg/min):<br>0-30: 10.2<br>30-60: 8.6<br>60-120: 8.8<br>120-180: 8.9	N/A	N/A
**LARGE**											
Guard *et al.*, 2019	2	33.5	3	Pitbull Terrier, Labrador Retriever	N/A	N/A	N/A	N/A	N/A	Faeces (µg/mg lyophilized)	CA: 0.2<br>CDCA: 0.1<br>LCA: 0.5<br>DCA: 1.5<br>UDCA: 0.01<br>BA I: 0.3<br>BA II: 2.0<br>Total: 2.3
**UNCLASSIFIED**											
Blake *et al.* 2019	34	N/A	4<br>(1-12)	Mixed Breed, German Shepherd, Miniature Schnauzer, Mastiff, Bull Terrier, Chinese Crested dog, Dachshund, English Cocker Spaniel, Husky, Miniature Poodle, Vizsla, Weimaraner	Maintenance diet	N/A	N/A	N/A	N/A	Faeces (µg/mg lyophilized faeces)	CA: 0.2<br>CDCA: 0.1<br>LCA: 1.0<br>DCA: 4.2<br>UDCA: 0.01<br>BA I: 0.4<br>BA II: 5.1<br>Total: 6.0
Jergens *et al.*, 2019	10	17.2<br>(5-37)	5.4<br>(2-9)	N/A	N/A	N/A	N/A	N/A	N/A	Faeces (µg/mg lyophilized faeces)	CA: 0.5<br>LCA: 0.4<br>BA I: 1.0<br>BA II: 2.5<br>Total: 3.5
Manchester *et al.*, 2019	8	19<br>(8.5-48)	3<br>(1.5-6)	N/A	N/A	GC-MS	N/A	N/A	N/A	Faeces (µg/mg lyophilized faeces)	Total unconjugated BA: 5.8 (1.2-14.8)<br>BA I: 0.3 (0-13.6)<br>BA II: 3.1 (1.2-12.2)
Herstad *et al.*, 2018	8	19.1<br>(10.3-28)	5.1<br>(1.5-8)	English Springer Spaniel, Small Münsterländer, Eurasier, Irish Setter, Mixed Breed, English Setter, English Cocker Spaniel, German Shorthaired Pointer	Commercial dry food	LC-MS/MS	N/A	N/A	N/A	Faeces (µg/g lyophilized faeces)	CA: 50<br>CDCA: 48<br>DCA: 60<br>LCA: 70<br>BA I: 98<br>BA II: 130<br>Total: 228

References	N=	Weight (kg)	Age (years)	Breed	Type of food	Methods	Saliva	Gastric juice	Pancreatic juice	Bile	
										Place of measure	Composition
Nagahara *et al.*, 2018	N/A	N/A	N/A	N/A	N/A	N/A	N/A	N/A	N/A	N/A	Mucin:<br>13.1 (10.7-21.7) mg/dL<br>Cholesterol:<br>97 (61-114) mg/dL<br>Bile flow: 19-36 mL/d/kg<br>Total bile:<br>1.6-2.9 mmol/d/kg<br>Concentration:<br>40-90 mmol/L
Ricci *et al.*, 2018	75	8-42	0.8-15	Mixed Breeds, pure breeds	N/A	Automatic biochemistry analyzer	Ca:<br>7.7 ± 2.5 mg/dL<br>P:<br>4.9 ± 2.5 mg/dL<br>LDH: 2690 ±<br>2216 UI/L<br>Amylase:<br>35.9 ± 41 UI/L<br>Lysozyme:<br>3.1 ± 4.4 mg/L	N/A	N/A	N/A	N/A
Sanguansermsri *et al.*, 2018	7	N/A	1-3	Mixed breeds	N/A	SDS-PAGE<br>LC-MS/MS	Amylase:<br>6.4 log2<br>Mucin-5:<br>8.7 log2<br>Mucin-7:<br>11.3 log2<br>Mucin-16: 7 log2<br>Mucin-19:<br>10.6 log2	N/A	N/A	N/A	N/A
Schmidt *et al.*, 2018	19	13.2<br>(6.5-30)	5<br>(2-15)	N/A	Dry food<br>(protein 30%, fat 18%, fibers 3%)	GC-TOF-MS	N/A	N/A	N/A	Faeces<br>(µg/mg lyophilized faeces)	BA I: 1<br>BA II: 6.5<br>Total BA: 7.5
Iacopetti *et al.*, 2017	29	16.5<br>(6-42.8)	1.5<br>(0.8-2)	Mixed Breeds, Flat Coat Retrievers, Whippets, American Staffordshire, Breton, Great Dane, German Shepherd, Basenji	Dry commercial diet	No eat 12h prior to sampling. Water remove 1h before<br><br>Automatic biochemistry analyzer	Ca:<br>7.5 ± 1.7 mg/dL<br>P:<br>3.6 ± 1.7 mg/dL<br>LDH: 2394.9 +<br>1669 UI/L<br>Amylase:<br>27.2 ± 19.2 UI/L<br>Lysozyme:<br>3.2 ± 4.1 mg/L	N/A	N/A	N/A	N/A
Kakimoto *et al.*, 2017	7	9.5-<br>11.3	3-4	Beagles	N/A	Samples from gallbladder contents were obtained by use of percutaneous ultrasound-guided cholecystocentesis or during cholecystectomy or necropsy.<br><br>HPLC	N/A	N/A	N/A	Gallbladder<br>(mmol/L)	TUDCA: 0.7 (0.4-0.9)<br>UDCA: 0.1 (0-0.2)<br>GCA: 2.1 (1.1-3.5)<br>CA: 0.5 (0-0.7)<br>CDCA: 0 (0-0)<br>TLCA: 0.5 (0-1.8)<br>Total BA: 252.7<br>(215.8-303.4) mg/dL<br>Cholesterol: 73<br>(59-100) mg/dL<br>Phospholipids: 4.2<br>(3.790-5.6) mg/dL

References	N=	Weight (kg)	Age (years)	Breed	Type of food	Methods	Saliva	Gastric juice	Pancreatic juice	Bile	
										Place of measure	Composition
Sams *et al.*, 2016	N/A	N/A	N/A	N/A	N/A	N/A	N/A	Lipase:<br>13 590 U/organ<br>230 U/kg BW	N/A	N/A	N/A
Lavy *et al.*, 2012	37	N/A	1-6	14 Dachshunds, Jack Russel Terriers, Labrador Retriever	Dry commercial diet	No eat 12h prior to sampling. Water *ad libidum*	Ca: 116.3 ± 46 mg/L<br>Phosphate: 13.3 ± 11.5 mg/L<br>Na: 164 ± 82 mg/L<br>K: 1105 ± 312 mg/L	N/A	N/A	N/A	N/A
Durand, 2010	N/A	N/A	N/A	N/A	N/A	N/A	99 % water<br>1 % mucus	N/A	pH 7.4-8.3	N/A	N/A
Center, 2009	N/A	N/A	N/A	N/A	N/A	N/A	N/A	N/A	N/A	Gallbladder and liver	Gallbladder:<br>Water: 85-90 %<br>Cholesterol: 10-30 mM<br>Bilirubin: 1.5-7 mM<br>Lecithin: 8 mM<br>Proteins: 700 mg/dL<br>Fatty acids: 350 mg/dL<br>Bic: < 1 mM or 8 mM<br>Ca^{2+}/Ca total: 2-3 mM or 10-18 mM<br>Na^{+}: 280 mM<br>K^{+}: 10 mM<br>Cl^{-}:15 mM<br>Liver:<br>Water: 95-97 %<br>Cholesterol: 3 mM (2-5)<br>Bilirubin: 0.8 mM (0.3-0.8)<br>Lecithin: 1 mM<br>Proteins: 250 mg/gL<br>Fatty acids: 250 mg/dL<br>Bic: 45 mM<br>Total Ca^{2+}/Ca: 0.8-1.2 mM or 2-3 mM<br>Na^{+}: 165<br>K^{+}: 5<br>Cl^{-}: 90<br>Total:<br>Liver: 35 mM<br>Gallbladder: 310 mM
Moschetta *et al.*, 2005	N/A	N/A	N/A	N/A	N/A	N/A	N/A	N/A	N/A	N/A	Bile flow: 19-36 mL/d/kg<br>Total bile:<br>1.6-2.9 mmol/d/kg
Martinez, 2002	N/A	N/A	N/A	N/A	N/A	N/A	Daily average salivary output: 0.5 L	N/A	4-10 µl/min/kg BW	N/A	N/A

References	N=	Weight (kg)	Age (years)	Breed	Type of food	Methods	Saliva	Gastric juice	Pancreatic juice	Bile	
										Place of measure	Composition
Kararli, 1995	N/A	N/A	N/A	N/A	N/A	N/A	N/A	Basal secretion rate:<br>0.1 mEq/h<br>Fed:<br>39 mEq/h<br>Basal production rate:<br>0.3-1.5 mL/min	Secretion rate: 1-3 mL/min	Bile	Bile flow: 19-36 mL/d/kg<br><br>Bile:<br>Flow rate: 1.6-2.9 mmol/d/kg<br>Total: 40-90 mmol/L
Stevens & Hume, 1995	N/A	N/A	N/A	N/A	100 g meat	N/A	Submaxillary glands of dog secreted at rate 7.5 times greater than that of humans	N/A	Amount of juice: 1316 mL<br>Time of secretion: 252 min<br>Na_2CO_3: 59 %	N/A	N/A
Washizu *et al.*, 1994	N/A	N/A	N/A	N/A	N/A	N/A	N/A	N/A	N/A	Gallbladder	TCA: 72.8 %<br>TDCA: 20.3 %<br>TCDCA: 6.2 %
Washizu *et al.*, 1990	3	N/A	N/A	Mixed breeds	N/A	N/A	N/A	N/A	N/A	Gallbladder (mg/mL)	TUDCA: 0.1 ± 0.1<br>TCA: 37.6 ± 9.1<br>TCDCA: 3.1 ± 1.4<br>TDCA: 10.3 ± 1.5<br>TLCA: 0.1 ± 0.1<br>CA: 0.01 ± 0.01<br>GCA: 0.2 ± 0.1<br>GLCA: 0.1 ± 0.1<br>Total: 51.4 ± 10.3
Kienzle, 1988	16	N/A	N/A	N/A	N/A	N/A	N/A	N/A	Chymotrypsin activity (lactose meal): 1.5 ± 0.7 U/kg BW<br>Amylase: 2013 ± 2017 U/g wet weight (383-6625)	N/A	N/A
Alvaro *et al.*, 1986	3	N/A	N/A	Mixed breed	Fasting for 12h	HPLC	N/A	N/A	N/A	Bile	TCA: 81.4 ± 9.8 %<br>TDCA: 12.6 ± 1.4 %<br>TUDCA: 5.9 ± 4.2 %<br>Phosphatidylethanolamine: 5.5 ± 0.8 %<br>Phosphatidylcholine: 94.5 ± 8.2 %
Dressman, 1986	N/A	N/A	N/A	N/A	N/A	N/A	N/A	Basal secretion rate:<br>0.1 mEq/h<br>Peak (maximal histamine response): 39 ± 5 mEq/h<br>(0.5 mEq/kg/h)	Bic secretion:<br>basal: 23.9 ± 17.8 mEq/L<br><br>Secretin test: 60 ± 20.5 mEq/L<br>(2 ± 1.29 mEq/kg/h)	N/A	N/A

References	N=	Weight (kg)	Age (years)	Breed	Type of food	Methods	Saliva	Gastric juice	Pancreatic juice	Bile	
										Place of measure	Composition
Bickel & Kauffman, 1981	N/A	N/A	N/A	N/A	N/A	Gastric output measured for 40 min	N/A	Total gastric acid output: 5.47 ± 1.72 mEq H+ (8.2 mEq/h)<br>Bic: 323 ± 87.7 mm/l<br>Protein: 310 ± 32.5 mg	N/A	N/A	N/A
Noel-Jorand *et al.*, 1981	15	N/A	N/A	N/A	Royal soup (royal canin) + canned minced meat	Fasted with water *ad libidum* since 18h<br><br>Bicarbonate was determined by back-titration. Chloride concentration was determined by chloridometer. Calcium, magnesium and zinc concentrations were determined using an atomic absorption spectrophotometer.	N/A	N/A	Basal rate:<br>Proteins: 33.4 mg/mL<br>53.1 mg/h<br>Vol. proteins: 1.9 mL/h<br>Bic: 54.3 mg/mL<br>92.3 mg/h<br>Chloride: 86.9 mg/mL<br>189.1 mg/h<br>Ca: 4.5 mg/mL<br>7.3 mg/h<br>Mg: 0.4 mg/mL<br>0.6 mg/h<br>Zn: 0.2 mg/mL<br>0.3 mg/h<br><br>pH 7.8	N/A	N/A

References	N=	Weight (kg)	Age (years)	Breed	Type of food	Methods	Saliva	Gastric juice	Pancreatic juice	Bile	
										Place of measure	Composition
Tiscornia *et al.*, 1977	3	N/A	N/A	N/A	Canned meal	Pancreatic secretion was measured for flow rate at 20-min intervals, bicarbonate concentration (Van Slyke volumetric method) and output at 20 min, and protein con- centration (absorbance reading at 280 nm) and output at 20 min.	N/A	N/A	<u>Bic concentration</u> (mEq/L):<br>T_0: 56<br>T_{20}: 101<br>T_{40}: 132<br>T_{60}: 123<br>T_{80}: 110<br>T_{100}: 106<br>T_{120}: 100<br><u>Bic output</u> (mEq/20min):<br>T_0: 0.07<br>T_{20}: 0.56<br>T_{40}: 1.27<br>T_{60}: 0.8<br>T_{80}: 0.5<br>T_{100}: 0.42<br>T_{120}: 0.38<br><u>Flow rate</u> (ml/20min):<br>T_0: 1<br>T_{20}: 5<br>T_{40}: 9.4<br>T_{60}: 6<br>T_{80}: 4.5<br>T_{100}: 4<br>T_{120}: 3.8<br><u>Protein concentration</u> (mg/mL):<br>T_0: 29<br>T_{20}: 22<br>T_{40}: 13<br>T_{60}: 17<br>T_{80}: 21<br>T_{100}: 20<br>T_{120}: 21<br><u>Protein output</u> (mg/20min):<br>T_0: 39<br>T_{20}: 102<br>T_{40}: 108<br>T_{60}: 99<br>T_{80}: 82<br>T_{100}: 70<br>T_{120}: 65	N/A	N/A
Larmas & Scheinin, 1971	7	5.5-12.2	0.4-1.25	Mixed breed	Protein 29%<br>Fat 3.5%<br>Fibers 4%	N/A	Ca: 1.9 (1-2.75) mmol/L<br>K: 20.2 (14.1-24.8) mmol/L<br>Na: 74.1 (42-99.8) mmol/L<br>Total P: 3.1 (1.2-5.9) mmol/L	N/A	N/A	N/A	N/A

References	N=	Weight (kg)	Age (years)	Breed	Type of food	Methods	Saliva	Gastric juice	Pancreatic juice	Bile	
										Place of measure	Composition
Patten, Richards, & Pope, 1971	8	6.1-19.1	N/A	N/A	Canine maintenance science diet	Measures have been done in pancreas directly after dog sacrifice	N/A	N/A	In pancreas:<br>Trypsinogen:<br>9.4 ± 0.5 U/mg protein<br>Chymotrypsinogen:<br>2.1 ± 0.1 U/mg protein<br>Lipase:<br>26.1 ± 3.3 U/mg proteins<br>Amylase:<br>49.2 ± 8.3 U/mg proteins	N/A	N/A
Nakayama, 1969	N/A	N/A	N/A	N/A	N/A	N/A	N/A	N/A	N/A	Gallbladder	Total solids:<br>196.6 ± 122.9 g/L<br>Cholesterol:<br>1.37 ± 0.75 g/L<br>Fatty acids: 0.25 ± 0.23 g/L<br>Monoacylglycerol:<br>2 ± 3 g/L<br>Diacylglycerol: 2 ± 4 g/L<br>Total lipids:<br>193.5 ± 49.8 g/L<br>Phospholipids:<br>20.3 ± 15.4 g/L<br>Bile flow: 19-36 mL/d/kg<br>Bile:<br>Total: 1.6-2.9 mmol/d/kg<br>Concentration:<br>40-90 mmol/L

References	N=	Weight (kg)	Age (years)	Breed	Type of food	Methods	Saliva	Gastric juice	Pancreatic juice	Bile	
										Place of measure	Composition
Altman & Dittmer 1968	N/A	N/A	N/A	N/A	N/A	N/A	N/A	N/A	N/A	Gallbladder and liver	Gallbladder:<br>DM: 114-246<br>Salts: 79-150 g/L<br>Ca: 131 mmol/L<br>Iodine: 130-1130 x 10^{-6} g/L<br>Iron: 0.9-1.8 x 10^{-3} g/mL<br>Total P: 0.9-2.8 g/L<br>Choline total: 3.4-11.1 g/L<br>Cholesterol : 0.8-1 g/L<br>Fatty acids : 16-50 g/L<br>Total proteins : 1.9-5.2 g/L<br>Bilirubin : 0.9-1.7 g/L<br><br>Liver :<br>DM : 23-45<br>Salts : 5-24<br>Na : 1.9-3.6<br>Chloride : 70 (59-105)<br>Iodine : 130-1130 x 10^{-6} g/L<br>Iron : 18-160 x 10^{-3}<br>Mg : 1.8 (1.1-2.5)<br>Total P : 0.1-0.2<br>K : 5.1-6<br>Na : 168 (150-203)<br>Bic : 7-34<br>Choline total : 0.4-0.6<br>Cholesterol : 0-0.2<br>Fatty acids : 1.8-2.7<br>Total proteins : 1.3-2.1<br>Bilirubin : 0.4-0.6
Alexander 1965	9	N/A	N/A	N/A	Tinned meat, 4-6h before abattage	Bicarbonate: microdiffusion method of Conway 1957.<br>Chloride: Eel chloride meter<br>Sodium and potassium are estimated using means of Eel flame photometer.<br>Phosphate was estimated as described by King and Wootton	N/A	Bic: 5.0 ± 0.7 mEq/L<br>K: 28.0 ± 3.7<br>Na: 58 ± 9.1<br>Chloride: 149 ± 5.3<br>P: 24 ± 5.3	N/A	N/A	N/A

<u>Supplementary Table S3</u>: Overview of studies reporting digestibility of healthy adult dogs depending on body weight in the different compartments of the digestive tract.

References are organized according to dog sizes (small, medium, large, unclassified) in reverse chronological order. AHF: acid-hydrolyzed fat; Ca: calcium; CP: crude protein; Cu: copper; DM: dry matter; Fe: iron; IF: insoluble fiber; K: potassium; Mg: magnesium; Mn: manganese; N/A: not applicable; Na: sodium; OM: organic matter; P: phosphorous; SF: soluble fiber; TDF: total dietary fiber; Zn: zinc.

References	N=	Weight (kg)	Age (years)	Breed	Type of food	Food composition (%)					Digestion	Macronutrient digestibility (%)	Micronutrient digestibility (%)
						Proteins	Fat	Fibers	Metabolizable energy	Others			
**SMALL**													
Nery *et al.*, 2010	7	4-6.5	N/A	Miniature Poodle, Jack Russell, Miniature Schnauzer	Experimental diet	28.6	17.6	TDF: 7.8	5.2 kcal/g	DM: 91.5<br>Ash: 9.1<br>Carbohydrates: 28.4<br>Na: 0.4<br>K: 1.0	Total apparent digestibility	DM: 83.5<br>CP: 84.1<br>Fat: 95.5<br>Ash: 30.1<br>Energy: 87.8	N/A
Weber *et al.*, 2003	N/A	3.6 ± 0.6	1.25	Miniature Poodle	Dry expanded diet	39.2	16.3	TDF: 7.3	1594 kJ/100 g	DM: 92.7<br>Ash: 6.9<br>Carbohydrates: 23.0	Total apparent digestion	DM: 82.0<br>OM:85.5<br>CP: 81.5<br>Fat: 94.4<br>Gross energy: 85.5<br>Ash: 32.6<br>TDF: 38.7	N/A
**MEDIUM**													
Alexander *et al.*, 2019	12	10.35 ± 0.88	3.2 ± 0.8	Beagles	Experimental diet	38.6	17.3	TDF: 7.2	N/A	N/A	Total apparent digestion	DM: 85.5<br>OM: 89.3<br>CP: 88.2<br>Fat: 95.0<br>Energy: 89.7	N/A
Detweiler *et al.*, 2019	8	12.8 ± 1.7	4.6 ± 0.6	Beagles	No fiber diet	31.7	AHF: 11.9	TDF: 5.0<br>IF: 2.4<br>SF: 2.6	3.7 kcal/g	DM: 90.2<br>OM: 93.1	Total apparent digestion	DM: 85.4<br>OM: 90.1<br>CP: 85.8<br>Fat: 90.9<br>TDF: 37.8<br>Energy: 83.4	N/A
Donadelli & Aldrich, 2019	12	N/A	2-4	Beagles	Cellulose-enriched diet	29.1	7.6	TDF: 20.5<br>Crude fiber: 8.2	N/A	DM: 95.4<br>Ash: 6.0<br>Carbohydrates: 32.3	Total apparent digestion	DM: 77.2<br>OM: 80.8<br>CP: 87.6<br>Crude fat: 90.9<br>Gross energy: 81.8	N/A
Eisenhauer *et al.*, 2019	10	13.7	4.4 ± 0.3	Beagles	Experimental dry diet	21.9	9.8	TDF: 3.4<br>Crude fiber: 0.9<br>IF: 1.9<br>SF: 1.5	N/A	Ash: 5.6	Total apparent digestion	OM: 90.3<br>CP: 81.8<br>Crude fat: 95.5<br>TDF: -1.9	Ca: 5.9<br>P: 33.1<br>Na: 90.8<br>K: 93.7<br>Mg: 19.4<br>Cu: 11.1<br>Fe: 10.6<br>Mn: 43.0<br>Zn: 10.9
Nogueira *et al.*, 2019	N/A	18 ± 1.38	N/A	N/A	Experimental diet	30.8	AHF: 18.5	TDF: 12<br>IF: 8.9<br>SF: 3.2	Gross energy: 5.3 kcal/g	DM: 93.1<br>OM: 92.6<br>Ash: 7.3<br>Carbohydrates: 24.5	Total apparent digestion	DM: 81.6<br>OM: 86.4<br>CP: 87.2<br>TDF: 25.6<br>Digestible energy: 87.4	N/A

References	N=	Weight (kg)	Age (years)	Breed	Type of food	Food composition (%)					Digestion	Macronutrient digestibility (%)	Micronutrient digestibility (%)
						Proteins	Fat	Fibers	Metabolizable energy	Others			
Algya *et al.*, 2018	8	13	3.57 ± 0.29	Beagles	Commercial dry diet	24.07	AHF: 13.3	TDF: 9.6	3.24 kcal/g	DM: 93.33	Total apparent digestion	DM: 82.6<br>OM: 87.8<br>CP: 85.1<br>Fat: 92.1<br>Energy: 87.4	N/A
Pinna *et al.*, 2018	12	19.5 ± 6.2	3.6 ± 1.6	N/A	Dry diet	N/A	N/A	N/A	N/A	N/A	Total apparent digestion	DM: 85<br>CP: 88<br>Ca: 4<br>P: 40<br>Mg: 1<br>Na: 97<br>K: 95	Zn: 21<br>Mn: 19<br>Fe: 1<br>Cu: 37
Beloshapka *et al.*, 2016	48	10	3.4	Beagles	Experimental diet	30.07	AHF: 18.58	TDF: 5.7<br>Insoluble: 4.17<br>Soluble: 1.53	5.12 kcal/g	DM: 96.13<br>OM: 91.86	Total apparent digestion	DM: 83<br>OM: 88.5<br>CP: 82.9<br>Fat: 95.3<br>Energy: 88.8	N/A
Hendriks *et al.*, 2013	5	25.3 ± 4.6	1-5	Mixed Breeds	Commercial diet	N/A	N/A	N/A	N/A	N/A	Ileal digestion	DM: 75.1<br>OM: 79.4<br>CP: 76.2<br>Crude fat: 96.5<br>Carbohydrates: 88.9	Arginine: 87.3<br>Histidine: 61.4<br>Isoleucine: 77.7<br>Leucin: 79.4<br>Lysine: 76.8<br>Methionine: 82.6<br>Phenylalanine: 80.3<br>Threonine: 62<br>Valine: 72.3<br>Alanine: 77.7<br>Aspartate: 67<br>Cysteine: 56.5<br>Glutamate: 81.6<br>Glycine: 73.1<br>Proline: 78.8<br>Serine: 69<br>Tyrosine: 77.2
											Total apparent digestion	DM: 81.2<br>OM: 85.3<br>CP: 81.9<br>Crude fat: 92.4<br>Carbohydrates: 95.5	Arginine: 91.7<br>Histidine: 78.7<br>Isoleucine: 80.6<br>Leucine: 84.9<br>Lysine: 81.6<br>Methionine: 83.8<br>Phenylalanine: 84.6<br>Threonine: 79<br>Valine: 79.6<br>Alanine: 81.6<br>Aspartate: 82<br>Cysteine: 75.3<br>Glutamate: 87.1<br>Glycine: 83.7<br>Proline: 88.2<br>Serine: 82.6<br>Tyrosine: 82.8
Beloshapka, Wolff, & Swanson, 2012	8	20 ± 0.5	3.5 ± 0.5	Mixed Breed	Experimental diet	N/A	N/A	N/A	N/A	N/A	Total apparent digestion	DM: 82.4<br>OM: 85.6<br>CP: 84.8<br>AHF: 95.2<br>Energy: 86.9	N/A

References	N=	Weight (kg)	Age (years)	Breed	Type of food	Food composition (%)					Digestion	Macronutrient digestibility (%)	Micronutrient digestibility (%)
						Proteins	Fat	Fibers	Metabolizable energy	Others			
Nery *et al.* 2010	6	14.4	4.8 ± 0.5	Standard Schnauzer	Experimental dry diet	28.6	17.6	TDF: 7.8	5.2 kcal/g	DM: 91.5<br>Ash: 9.1<br>Carbohydrates: 28.4<br>Na: 0.4<br>K: 1.0	Total apparent digestion	DM: 85.2<br>CP: 86.2<br>Fat: 96<br>Ash: 34.9<br>Energy: 89.2	N/A
	6	27.2		Giant Schnauzer								DM: 86.4<br>CP: 87.6<br>Fat: 95.8<br>Ash: 37.2<br>Energy: 90.1	N/A
	8	23.1		German Shepherd								DM: 85.5<br>CP: 85.8<br>Fat: 95.1<br>Ash: 35.8<br>Energy: 89.2	N/A
Zentek *et al.*, 2004	8	12.4 ± 2.2	1-11	Beagles	Commercial diet	31.0	14.6	2.5	17.2 MJ	N/A	Total apparent digestion	DM: 79.3<br>CP: 79.3<br>Fat: 93.5	N/A
	3	27.4 ± 2.7	6	German Shorthair Pointers	Commercial diet	30.5	15.1	2.3	16.8 MJ	N/A	Total apparent digestion	DM: 78.6<br>CP: 80.6<br>Fat: 96.0	N/A
Flickinger *et al.*, 2003	4	12 ± 1.3	3	Beagles	Commercial diet	N/A	N/A	N/A	N/A	N/A	Ileal digestion	DM: 60.3<br>OM: 68<br>CP: 59.4<br>Fat: 92.3	N/A
											Total apparent digestion	DM: 60.3<br>OM: 87.0<br>CP: 82.9<br>Fat: 94.7	N/A
Weber *et al.*, 2003	6	12.6 ± 0.9	1.25	Medium Schnauzers	Dry expanded diet	39.2	16.3	TDF: 7.3	1594 kJ/100 g	DM: 92.7<br>Ash: 6.9<br>Carbohydrates: 23	Total apparent digestion	DM: 84.2<br>OM: 86.3<br>CP: 82.6<br>Fat: 96.2<br>Gross energy: 86.9<br>Ash: 66.0<br>TDF: 48.9	N/A
	6	23.3 ± 1.3	1.25	Giant Schnauzers								DM: 85.7<br>OM: 88.9<br>CP: 85.9<br>Fat: 95.0<br>Gross energy: 89.3<br>Ash: 37.3<br>TDF: 53.1	N/A
Zentek, Kaufmann, & Pietrzak, 2002a	4	N/A	N/A	Beagles	Dry diet	28.3	17	Crude fiber: 3.3	N/A	DM: 93.9<br>Ash: 6.5<br>Carbohydrates: 38.8<br>Ca: 1.3<br>Phosphorous: 0.8	Total apparent digestion	DM: 32.8<br>OM: 85.3<br>CP: 79.2	N/A
				German shorthair								DM: 28.8<br>OM: 87.0<br>CP: 80.9	N/A
				German shepherd								DM: 28.5<br>OM: 86.4<br>CP: 79.9	N/A
Zentek, Marquart, & Pietrzak, 2002b	4	11.7 ± 2.2	"adult"	Beagles	Experimental diet	36.6	24.4	Crude fiber: 4.9	N/A	DM: 94.5<br>Ash: 4.0<br>Carbohydrates: 24.6	Total apparent digestion	DM: 85.0<br>CP: 82.5<br>Fat: 96.2<br>Crude fiber: 61.8	N/A

References	N=	Weight (kg)	Age (years)	Breed	Type of food	Food composition (%)					Digestion	Macronutrient digestibility (%)	Micronutrient digestibility (%)
						Proteins	Fat	Fibers	Metabolizable energy	Others			
Bednar *et al.*, 2000	4	25.5 ± 3.9	4 (3-5)	N/A	Beef and bone meal	24.5	14.4	TDF: 9.6	N/A	Arginine: 1.4<br>Histidine: 0.6<br>Isoleucine: 1<br>Leucine: 2.2<br>Lysine: 1.2<br>Methionine: 0.5<br>Phenylalanine: 1.2<br>Threonine :0.9<br>Valine: 1.1<br>Alanine: 1.3<br>Cysteine: 2.3<br>Glutamate: 0.8<br>Glycine: 4.7<br>Proline: 1.0<br>Serine: 1.6<br>Tyrosine: 0.8	Ileal digestion	DM: 65.7<br>OM: 75.6<br>CP: 68.3<br>Fat: 89.3<br>TDF: 17.8	Arginine: 79.4<br>Histidine: 68.5<br>Isoleucine: 73.1<br>Leucine: 78.5<br>Lysine: 74.8<br>Methionine: 84.7<br>Phenylalanine: 74.8<br>Threonine: 58.5<br>Valine: 70<br><br>Alanine: 77.8<br>Aspartate: 53.8<br>Cysteine: 40.0<br>Glutamate: 78.9<br>Glycine: 74.3<br>Proline: 76.7<br>Serine: 65.1<br>Tyrosine: 54.4
											Total apparent digestion	DM: 78.6<br>OM: 86.5<br>CP: 82.4<br>Fat: 90.1<br>TDF: 44.2	N/A
Fahey *et al.*, 1990b	30	17.1	2-6	English Pointers	Experimental diet	33.2	N/A	TDF: 5.4<br>Crude fiber: 1.1	N/A	DM: 93.1<br>OM: 92.4<br>Ash: 7.6<br>Starch: 31.1	Total apparent digestion	DM: 90.4<br>OM: 93.4<br>Crude fiber: 31.3<br>TDF: 52.3	N/A
**LARGE**													
Weber *et al.*, 2003	6	46.3 ± 1	N/A	Great Danes	Dry expanded diet	39.2	16.3	TDF: 7.3	1594 kJ/100 g	DM: 92.7<br>Ash: 6.9<br>Carbohydrates: 23	Total apparent digestion	DM: 85.4<br>OM: 88.4<br>CP: 84.8<br>Fat: 95.8<br>Gross energy: 89.9<br>Ash: 41.7<br>TDF: 52.5	N/A
**UNCLASSIFIED**													
Propst *et al.*, 2003	7	N/A	"adult"	N/A	Kibbles	32.7	23.5	N/A	N/A	DM: 92.9<br>OM: 93.3	Ileal digestion	DM: 60<br>OM: 67.5<br>CP: 51.3<br>Fat:93.7	N/A
											Total apparent digestion	DM: 83<br>OM: 87.4<br>CP: 81.7<br>Fat: 94.8	N/A
Meyer *et al.*, 1999	66	4.2-52.5	1.5-9.7	10 breeds	Dry diet	29.4	13.8	TDF: 3.4<br>IF: 2.8<br>SF: 0.5	N/A	DM: 91.5	Total apparent digestion	OM: 88.9<br>CP: 86.0<br>Fat: 93.8<br>Fiber: 30.6	N/A
Fahey *et al.*, 1990a	N/A	N/A	N/A	N/A	N/A	33.3	N/A	TDF: 6.1<br>Crude fiber: 0.7	N/A	DM: 93.8<br>OM: 92.5<br>Starch: 31.3	Total apparent digestion	DM: 87.6<br>OM: 90.2<br>TDF: 44.4<br>Crude fiber: 9.9	N/A

Supplementary Table S4: Overview of studies reporting transit time of healthy adult dogs depending on body weight (i.e. gastric emptying. small intestine transit time, orocaecal transit time, large intestine transit time and total transit time). References are organized according to dog sizes (small, medium, large, unclassified) in reverse chronological order. [1]: Mean TTT was calculated from the recovery from feces of ingested colored plastic beads. [2]: Minimum OCTT was defined as the time from ingestion of the meal to the time when the total sulfapyridine concentration was detected in plasma. LITT was estimated as the difference between total transit time and orocaecal transit time. [3]: Radiographs were taken each 30 min. OCTT was define as the time when first marker reach colon. ∅: diameter; GET: gastric emptying time; LITT: large intestinal transit time; N/A: not applicable; OCTT: orocaecal transit time; RM: radiopaque markers; SI: small intestine; SITT: small intestinal transit time; SM: sulfasalazine method; TTT: total transit time.

References	N=	Weight (kg)	Age (years)	Breed	Food				Methods	Gastric emptying time (h)		Mean transit time (h)			
					Type of food	Protein (%)	Fat (%)	Fiber (%)		Fasted	Fed	SITT	OCTT	LITT	TTT
**SMALL**															
Hernot *et al.* 2006	6	3.8 ± 1	5.5-6	Miniature Poodle	Dry diet	24.7	16	7.7	OCTT and LITT: Sulfasalazine method[2] TTT: plastic beads[1]	N/A	N/A	N/A	13.8 ± 0.8	9.1 ± 1.1	22.9 ± 0.9
Weber 2006	N/A	N/A	N/A	Miniature Poodle	N/A	N/A	N/A	N/A	SITT: Radiopaque markers (RM) OCTT: RM + sulfasalazine method (SM) TTT: plastic beads	N/A	N/A	2.0 ± 0.3	RM: 2.5 ± 0.4 SM: 2.2 ± 0.5	N/A	22.9 ± 0.9
Hernot *et al.* 2005	1	6.3	N/A	Dachshund	Dry expanded diet	24.7	16	7.7	Plastic beads[1]	N/A	N/A	N/A	N/A	N/A	24.0
	2	7.6 (7.2-8)	N/A	Cairn Terrier		24.7	16	7.7		N/A	N/A	N/A	N/A	N/A	25 ± 5.9 (21-29)
	6	3.6 (3-5.2)	N/A	Miniature Poodle		24.7	16	7.7		N/A	N/A	N/A	N/A	N/A	24 ± 2 (22-26)
	3	6.5 (6.2-6.8)	N/A	Cavalier King Charles		24.7	16	7.7		N/A	N/A	N/A	N/A	N/A	31 ± 2.1 (29-33)
Weber *et al.* 2003	6	3.6 ± 0.6	1.25	Miniature Poodle	Dry expanded diet	39.2 ± 0.4	16.3 ± 0.2	9.8 ± 0.5	OCTT: Sulfasalazine method[2]	N/A	N/A	N/A	2.2 ± 0.5	N/A	N/A
Weber *et al.* 2002	6	N/A	1.25	Miniature Poodle	Dry expanded diet	39.2 ± 0.4	16.3 ± 0.2	9.8 ± 0.5	Radiopaque markers[3]	N/A	T_{50} = 6.4 ± 0.5 T_{100} (prediction): 18.4	2	2.5	N/A	N/A
**MEDIUM**															
Koziolek *et al.* 2019	6	13-18	2-4	Beagles	Dry or canned food	N/A	N/A	N/A	Telemetric motility capsule (SmartPill®)	T_{100}: 0.6 ± 0.4	T_{100}: 2.9 ± 0.9	Fasted: 1.4 ± 0.6 Fed: 1.9 ± 0.3	N/A	Fasted: 25.4 ± 3.3 Fed: 28.2 ± 4.7	Fasted: 27.3 ± 3.3 Fed: 33.0 ± 4.1
Boscan *et al.* 2013	12	27.2 (23-30)	1.75 (1.6-2)	Treeing Walker Hound	Dry food	N/A	N/A	N/A	Wireless motility capsule	N/A	T_{100}: 12 (5.3-16)	Fed: 2.8 (2-3.8)	N/A	N/A	N/A
Mahar *et al.* 2012	14	8-14	1-5	Beagles	N/A	25.5	18	2.8	Bravo pH telemetry system attached to the gastric mucosa	T_{100}: 0.4 ± 0.5	T_{100}: 11.4 ± 5.9	N/A	N/A	N/A	N/A
Sagawa *et al.* 2009	16	12 (9.5-14.5)	4.7 (3-6)	Beagles	2 liquid meals then 10 or 200 g kibbles	N/A	N/A	N/A	Bravo pH telemetry system	T_{100}: 1.4	T_{100}: 10 g: 9.4 200 g: 20	N/A	N/A	N/A	

References	N=	Weight (kg)	Age (years)	Breed	Food				Methods	Gastric emptying time (h)		Mean transit time (h)			
					Type of food	Protein (%)	Fat (%)	Fiber (%)		Fasted	Fed	SITT	OCTT	LITT	TTT
Hernot *et al.* 2006	6	12.9 ± 2.1	N/A	Standard Schnauzer	Dry diet	24.7	16	7.7	OCTT and LITT: Sulfasalazine method[2] TTT: plastic beads	N/A	N/A	N/A	14.3 ± 1	18.5 ± 3	32.8 ± 2.7
	6	23.9 ± 2.8	N/A	Giant Schnauzer		24.7	16	7.7		N/A	N/A	N/A	15.7 ± 1.3	39.4 ± 1.6	55.1 ± 1.3
Weber 2006	N/A	N/A	N/A	Standard Schnauzer	N/A	N/A	N/A	N/A	SITT: Radiopaque markers OCTT: RM + sulfasalazine method TTT: Plastic beads	N/A	N/A	1.8 ± 0.2	RM: 3.0 ± 0.4 SM: 2.4 ± 0.4	N/A	32.8 ± 2.7
	N/A	N/A	N/A	Giant Schnauzer	N/A	N/A	N/A	N/A		N/A	N/A	1.08 ± 0.3	RM: 2.9 ± 0.6 SM: 2.5 ± 0.6	N/A	55.1 ± 1.3
Hernot *et al.* 2005	1	13.8	N/A	French Bulldog	Dry expanded diet	24.7	16	7.7	Plastic beads	N/A	N/A	N/A	N/A	N/A	27
	4	11.2 (8.8-13.6)	N/A	English Cocker Spaniel		24.7	16	7.7	N/A	N/A	N/A	N/A	N/A	N/A	32 ± 6.7 (24-40)
	6	12.8 (10-14.9)	N/A	Standard Schnauzer		24.7	16	7.7	N/A	N/A	N/A	N/A	N/A	N/A	33 ± 6.6 (23-41)
	5	28.9 (26.3-33)	N/A	Labrador Retriever		24.7	16	7.7	N/A	N/A	N/A	N/A	N/A	N/A	35 ± 6.8 (25-43)
	6	23.7 (19.7-28.1)	N/A	Giant Schnauzer		24.7	16	7.7	N/A	N/A	N/A	N/A	N/A	N/A	55 ± 3.1 (51-59)
	6	26.9 (22.9-29.8)	N/A	German Shepherd		24.7	16	7.7	N/A	N/A	N/A	N/A	N/A	N/A	40 ± 6 (31-49)
Weber *et al.* 2003	6	12.6 ± 0.9	1.25	Standard Schnauzer	Dry expanded diet	39.2 ± 0.4	16.3 ± 0.2	9.8 ± 0.5	Sulfasalazine method[2]	N/A	N/A	N/A	2.4 ± 0.4	N/A	N/A
	6	23.3 ± 1.3	1.25	Giant Schnauzer						N/A	N/A	N/A	2.5 ± 0.6	N/A	N/A
Weber *et al.* 2002	6	N/A	1.25	Standard Schnauzer	Dry expanded diet	39.2 ± 0.4	16.3 ± 0.2	9.8 ± 0.5	Radiopaque markers[3]	N/A	T_{100}: 16.6 T_{50}: 6.5 ± 1.2	1.75	3.1	N/A	N/A
	6	N/A	1.25	Giant Schnauzer		39.2 ± 0.4	16.3 ± 0.2	9.8 ± 0.5		N/A	T_{100}: 19.8 T_{50}: 7.8 ± 0.7	1.75	2.9 ± 0.6	N/A	N/A
Cullen & Kelly 1996	14	15-20	N/A	Mixed Breeds	Labelled chicken liver	65	33	N/A	Labelled chicken liver	N/A	Solids: T_{50}: 4.1 ± 0.2 Liquids: T_{50}: 2.5 ± 0.2	N/A	N/A	N/A	N/A
Carrière *et al.* 1993	5	9.5-16	2-3	Beagles	Liquid test meal	N/A	N/A	N/A	Phenol red marker	N/A	T_{50}: 1.25 ± 0.13	N/A	N/A	N/A	N/A

References	N=	Weight (kg)	Age (years)	Breed	Food				Methods	Gastric emptying time (h)		Mean transit time (h)			
					Type of food	Protein (%)	Fat (%)	Fiber (%)		Fasted	Fed	SITT	OCTT	LITT	TTT
Papasouliotis *et al.* 1993	7	10-15	"varying"	N/A	Canned food + 7.7 g fibers	7.6	4.2	1.1-1.8	Breath test	N/A	N/A	N/A	Canned food: 1.8 (0.8-2.3)<br>CF + wheat bran: 1.9 (0.9-3.4)<br>CF + guar gum: 1.8 (1.3-3.3)	N/A	N/A
Arnbjerg 1992	N/A	20-30	N/A	N/A	Commercial dry food	N/A	N/A	N/A	Radiography	N/A	T_{100}: 15 ± 1	N/A	N/A	N/A	
Fahey *et al.* 1992	5	12.6-23.4	N/A	English pointers	Commercial dry food	33.1	N/A	6.2	Chrome oxide	N/A	N/A	N/A	N/A	N/A	24.6
Neri *et al.* 1991	5	14-16	N/A	Mixed breeds	Canned food	N/A	N/A	N/A	Labelled markers	N/A	N/A	Ileum-colon:<br>T_{50} fasting: 0.8 ± 0.1<br>T_{50} early postprandial period: 0.4 ± 0.2<br>T_{50} late PP: 0.2 ± 0.1	N/A	Early postprandial phase: 1.0 ± 0.2<br>Late PP: 1.5 ± 0.2	N/A
Fahey *et al.* 1990a	5	17.1 (10.2-20.8)	2-6	English pointers	Commercial dry food	33.2		5.4	Chrome oxide	N/A	N/A	N/A	N/A	N/A	19.1
Fahey *et al.* 1990b	5	12.6-23.4	N/A	English pointers	Commercial dry food	33.3		6.1	Chrome oxide	N/A	N/A	N/A	N/A	N/A	32.7
Hornof *et al.* 1989	6	12	N/A	Beagles	Beef baby food + kibble	N/A	N/A	N/A	Radio scintigraphy	N/A	T_{100}: 4.9 ± 2.0	N/A	N/A	N/A	N/A
Gupta & Robinson 1988	3	15-20	"adult"	Beagles	Fasted or 100 mL water	N/A	N/A	N/A	Thomas' cannula	T_{100}: 0.7	T_{50} water: 0.6-0.7<br>T_{100} water: 0.9-1.1	N/A	N/A	N/A	N/A
Lui *et al.* 1986	4	10-20	N/A	Beagles	Liquid meal	N/A	N/A	N/A	Heidelberg capsule	N/A	1.7 ± 0.5	N/A	N/A	N/A	N/A
Miyabayashi *et al.* 1986	5	12.4-13.7	2-8	Beagles	N/A	N/A	N/A	N/A	Barium sulfate contrast	T_{100}: 1.3 ± 0.3 (0.5-2)	N/A	1.2 ± 0.3 (0.5-2)<br>SI emptying time: 3.6 ± 0.4 (3-5)	N/A	N/A	N/A
Meyer *et al.* 1985	6	20-25	N/A	Mixed breeds	Radiolabeled steak + liver	N/A	N/A	N/A	Radiolabeled meal	N/A	T_{100}: 3	N/A	N/A	N/A	N/A
Youngberg *et al.* 1985	4	N/A	N/A	Beagles	N/A	N/A	N/A	N/A	Heidelberg capsule	T_{100}: 1.2 ± 0.4	T_{100}: > 6	N/A	N/A	N/A	N/A
Ehrlein & Pröve 1982	5	13-17	N/A	Beagles	Low. medium and high viscosity	N/A	N/A	N/A	N/A	N/A	T_{50}:<br>L: 0.1<br>M: 0.5 ± 0.2<br>H: 0.7 ± 0.2	N/A	N/A	N/A	N/A

References	N=	Weight (kg)	Age (years)	Breed	Food				Methods	Gastric emptying time (h)		Mean transit time (h)			
					Type of food	Protein (%)	Fat (%)	Fiber (%)		Fasted	Fed	SITT	OCTT	LITT	TTT
Sarr & Kelly 1980	6	14-16	N/A	Mixed breeds	A: liquid meal (perfusion)<br>B: liquids + solids<br>C: solid meal (50 solid nylon spheres)	N/A	N/A	N/A	Mean liquid or solid transit time = T_{50}	N/A	N/A	Jejunal segment:<br>A:<br>Fed 30 min: 6.7 ± 1.3<br>120 min: 7.2 ± 1.6<br>B:<br>Fed 30 min: 6.3 ± 1.2<br>120 min: 6.5<br>C:<br>Fed 30min: 147 ± 12<br>120min: 172 ± 13	N/A	N/A	N/A
Theodorakis 1980	6	14.1 (12.2-15.3)	"mature"	Beagles	255g canned food	N/A	N/A	N/A	Scintigraphy	N/A	T_{50}: 1.3 ± 0.4	N/A	N/A	N/A	N/A
Hinder & Kelly 1977	4	13-15	N/A	Mixed breeds	Liver	N/A	N/A	N/A	Radio scintigraphy	N/A	around 2	N/A	N/A	N/A	N/A
**LARGE**															
Coleman *et al.* 2019	9	43 (32-52)	2 (1-3.5)	3 Great Dane, 2 German Shepherd, Mastiff, Saint Bernard Cross, Rhodesian Ridgeback and Bernese Mountain	Dry food diet	N/A	N/A	N/A	Wireless motility capsule (SmartPill®)	N/A	19 (8.1-20.5)	N/A	N/A	N/A	N/A
Balsa *et al.* 2017	10	39.8 (21.2-68.5)	1.5 (0.4-4)	Great Dane, 2 German Shepherd, 2 Standard Poodle, Doberman Pinscher, Irish Wolfhound and Mixed breed	Standardized highly digestible intestinal diet	N/A	N/A	N/A	Wireless motility capsule	N/A	T_{100}: 7.2 (5.1-22.8)	N/A	20.7 (14-51.2)	N/A	32.8 (20.1-57.8)
Gazzola *et al.* 2017	10	55.3	2.5-6.5	8 Great Dane, 2 Great Dane/Weimaraner crossbreed	N/A	N/A	N/A	N/A	Wireless motility capsule	2 ± 0.9	N/A	1.9 ± 0.8	N/A	13.6 ± 7.7	18.2 ± 8.4
Warrit *et al.* 2017b	42	31.4 (11.8-63.8)	4 (1-11)	Mixed breeds	Dry food	N/A	N/A	N/A	Wireless motility capsule	N/A	17.6 (9.7-80.8)	4.2 (2.9-5.4)	N/A	25 (1.1-49.1)	N/A
Hernot *et al.* 2006	6	51.5 ± 2.1	N/A	Great Dane	Dry diet	24.7	16	7.7	OCTT and LITT: Sulfasalazine method[2]<br>TTT: Plastic beads	N/A	N/A	N/A	14.0 ± 1.1	29.3 ± 1.3	43.3 ± 0.4
Weber 2006	N/A	N/A	N/A	Great Dane	N/A	N/A	N/A		SITT: Radiopaque markers<br>OCTT: RM + sulfasalazine method<br>TTT: Plastic pellets	N/A	N/A	2.3 ± 0.1	RM: 3.0 ± 0.2<br>SM: 2.7 ± 0.6	N/A	43.2
Hernot *et al.* 2005	2	44.2 (43-45.3)	N/A	Newfoundland	Dry expanded diet	24.7	16	7.7	Plastic beads	N/A	N/A	N/A	N/A	N/A	45 ± 4.7 (42-48)
	1	58.6	N/A	Saint Bernard	Dry expanded diet	24.7	16	7.7		N/A	N/A	N/A	N/A	N/A	45.0
	7	53.6 (47.5-64.6)	N/A	Great Dane	Dry expanded diet	24.7	16	7.7		N/A	N/A	N/A	N/A	N/A	43 ± 2.6 (38-46)

References	N=	Weight (kg)	Age (years)	Breed	Food				Methods	Gastric emptying time (h)		Mean transit time (h)			
					Type of food	Protein (%)	Fat (%)	Fiber (%)		Fasted	Fed	SITT	OCTT	LITT	TTT
Weber *et al.* 2003	6	46.3 ± 1	1.25	Great Danes	Dry expanded diet	39.2 ± 0.4	16.3 ± 0.2	9.8 ± 0.5	Sulfasalazine method (total concentration in plasma)	N/A	N/A	N/A	2.7 ± 0.6	N/A	N/A
Weber *et al.* 2002	6	N/A	1.25	Great Danes	Dry expanded diet	39.2 ± 0.4	16.3 ± 0.2	9.8 ± 0.5	Radiopaque markers[3]	N/A	16.8<br>T_{50}: 6.4 ± 1.1	2.25	3.0 ± 0.2	N/A	N/A
Wyse *et al.* 2001	6	27-33	"adult"	N/A	36g bread, 200 mL skim milk + 5g sunflower margarine	N/A	N/A	N/A	Breath test	N/A	3.4 ± 0.5	N/A	N/A	N/A	N/A
**UNCLASSIFIED**															
Lidbury *et al.* 2012	8	29.1 (22.1-41)	1.8 (0.8-11)	Mixed Breeds, Flat Coated Retriever, German Shepherd, Golden Retriever	N/A	N/A	N/A	N/A	Wireless motility capsule	12 (10.4-22)	N/A	3.05 (1.5-4.8)	N/A	23.3 (10.7-40.8)<br>Small intestine + colon: 27.3 (12.4-43.1)	45.6 (36.6-54.9)
Boillat *et al.* 2010	31	41.2 (19.6-81.2)	1-10	Cross Breed, Great Dane, Rottweilers, Labrador Retriever, Alaskan Malamute, Australian Cattle Dog, Chesapeake Bay Retriever, Golden Retriever, Husky, Mastiff, Poodle, Rhodesian Ridgeback	Regular dry kibble diet	N/A	N/A	N/A	Wireless motility capsule	N/A	T_{100}: 6.8-15.0	1.6-3.7	N/A	7.1-42.9	21.6-57.4
Bourreau *et al.* 2004	24	25.96 (3.5-59.1)	4 ± 0.3	Miniature Poodle, Beagle, Schnauzer, Giant Schnauzer, Great Dane, Labrador Retriever, Argentine Dane	Commercial dry food	25.7	N/A	7.5	Sodium ^{13}C-octanoate breath test	N/A	25%: 1.4 ± 0.1<br>50%: 2.6 ± 0.1<br>75%: 4 ± 0.1	N/A	N/A	N/A	N/A
Bruce *et al.* 1999	10	20.2 (14-28)	0.7-5.5	Collie cross	Commercial high fiber diet	25.6	7	26.4	Barium impregnated polyethylene<br>S: Ø 1.5 mm<br>L: Ø 5 mm	N/A	N/A	N/A	N/A	90%:<br>S: 14 (10-19.3)<br>L: 16.6 (12-22)<br>Proximal:<br>S: 4.8<br>L: 3.2<br>Distal: S: 7.1<br>L: 8.3	S: 12<br>L: 11.5
Davies & Morris 1993	N/A	N/A	N/A	N/A	N/A	N/A	N/A	N/A	N/A	1.6	N/A	N/A	N/A	N/A	N/A
Hall *et al.* 1992	10	N/A	N/A	N/A	Dry food	N/A	N/A	N/A	Radiography	N/A	3.5 (1-6)	N/A	N/A	N/A	N/A
Hornof *et al.* 1989	5	5.4-35	N/A	Mixed breeds	N/A	N/A	N/A	N/A	Radio scintigraphy	N/A	T_{50}: 3.6	N/A	N/A	N/A	N/A
Dressman 1986	N/A	N/A	N/A	N/A	A: Liquids<br>B: 100 g steak + liver<br>C: 50g cubes of liver<br>D: 60 g steak + 30 g liver<br>E: 100g foie	N/A	N/A	N/A	N/A	N/A	A: 90 % emptied in 25 min<br>B: T_{50}: 1.5<br>C: T_{50}:1<br>D: T_{50}: 3<br>E: T_{50}:3	1.9 ± 0.3 (0.3-3.4)	N/A	N/A	N/A

References	N=	Weight (kg)	Age (years)	Breed	Food				Methods	Gastric emptying time (h)		Mean transit time (h)			
					**Type of food**	**Protein (%)**	**Fat (%)**	**Fiber (%)**		**Fasted**	**Fed**	**SITT**	**OCTT**	**LITT**	**TTT**
Burrows *et al.* 1985	N/A	26-32	N/A	N/A	Isocaloric commercial diet canned meat-based food (CM), dry cereal-based chow + water, dry cereal-based food (DC)	N/A	N/A		N/A	N/A	T_{50}: CM: 3.8 ± 0.6 DC + water: 2.5 ± 0.6 DC: 2.4 ± 0.6	N/A	N/A	N/A	N/A
Van den Brom & Happé 1985	27	N/A	N/A	N/A	Eggs, starch + glucose	N/A	N/A	N/A	Radio scintigraphy	N/A	T_{50}: 1.1 0.8-3.8 (95%)	N/A	N/A	N/A	N/A
Bueno *et al.* 1981	N/A	N/A	N/A	N/A	Canned food + 30 g fibers	N/A	N/A	N/A	Labelled markers Measures through a 200 cm jejunal segment 2-4h after feeding	N/A	N/A	Jejunal segment: 0.2 ± 0.4	N/A	N/A	N/A
Dozois *et al.* 1971	N/A	N/A	N/A	N/A	Liquid meal	N/A	N/A	N/A	N/A	N/A	T_{50}: 0.4	N/A	N/A	N/A	N/A

<u>Supplementary Table S5</u>: Overview of studies reporting gut microbiota composition in healthy adult dogs depending on body weight.

References are organized according to dog sizes (small, medium, large, unclassified) in reverse chronological order. CF: crude fiber; CFU: colony forming units; DGGE: denaturing gradient gel electrophoresis; DNA: deoxyribonucleic acid; FISH: fluorescence in situ hybridization; IF: insoluble fiber; N/A: not available; ND: not detected; qPCR: quantitative polymerase chain reaction; RNA: ribonucleic acid; SF: soluble fiber; TDF: total dietary fiber.

References	N=	Body weight (kg)	Age (years)	Breed	Food				Methods	Gut microbiota composition			
					Type of food	Protein (%)	Fat (%)	Fiber (%)		Stomach	Small intestine	Large intestine	Stools
SMALL													
You & Kim 2021	89		0.5-10	2 Greyhound,<br>6 Dachshund, 28 Maltese, 5 Bichon, 3 Yorkshire Terrier, 5 Chihuahua, 6 Pomeranian,<br>34 Poodle	Commercial diet	30	17	6	Illumina 16S rRNA sequencing MiSeq	N/A	N/A	N/A	*Firmicutes*: 41.9%<br>*Bacteroidetes*: 28.3%<br>*Fusobacteria*: 15.9%<br>*Proteobacteria*: 9.8%<br>*Actinobacteria*: 3.1%<br>*Deferribacteres*: 0.8%<br>*Tenericutes*: 0.5%
Reddy *et al.*, 2019	8	4.6	8	Maltese	Experimental diet	27	10	5	Illumina 16S rRNA sequencing MiSeq	N/A	N/A	N/A	Shannon index: 3.6<br>Chao1: 193.6<br>*Firmicutes*: 57.6%<br>whose *Streptococcus*: 3.8%<br>and *Turicibacter*: 2.6%<br>*Fusobacteria*: 2.6%<br>(*Fusobacterium* only)<br>*Proteobacteria*: 1.8%<br>whose *Anaerobiospirillum*: 0.2%
	8	3.8	8	Miniature Schnauzer		27	10	5		N/A	N/A	N/A	Shannon index: 3.7<br>Chao1: 192.7<br>*Firmicutes*: 80.2%<br>*Streptococcus*: 17.8%<br>*Turicibacter*: 0.4%<br>*Proteobacteria*: 1.5%<br>*Succinivibrio*: 0.6%<br>*Fusobacteria*: 1.5%<br>(*Fusobacterium*: 1.5%)
	9	7.6	8	Poodle		27	10	5		N/A	N/A	N/A	Shannon index: 3.3<br>Chao1: 171.5<br>*Firmicutes*: 78.3%<br>*Streptococcus*: 9.4%<br>*Turicibacter*: 2.5%<br>*Proteobacteria*: 1.1%<br>*Anaerobiospirillum*: 0.4%<br>*Fusobacteria*: 1.5%<br>(*Fusobacterium* only)
Omatsu *et al.*, 2018	20	N/A	0-11	Toy Poodle	N/A	N/A	N/A	N/A	16S rRNA barcoding analysis	N/A	N/A	N/A	*Firmicutes*: 30.3%<br>(12.4-90.1)<br>*Proteobacteria*: 14.8%<br>(0-98.6)<br>*Bacteroidetes*: 12.7%<br>(0-71.6)<br>*Fusobacteria*: 0.82%<br>(0-25.9)<br>*Enterobacteriaceae*: 24.2%<br>*Bacteroidaceae*: 19%<br>*Lachnospiraceae*: 13.4%
Kim *et al.*, 2017	24	4.3	3 (1-12)	Maltese, Yorkshire Terrier, Pomeranian, Poodle, Bichons Fries, White West Highland Terrier	Commercial diet	N/A	N/A	N/A	Illumina 16S rRNA sequencing	N/A	N/A	N/A	Shannon index: 2.2 ± 0.3<br>Chao1: 143.3 ± 23.8<br>Simpson index: 0.2 ± 0.1<br>*Firmicutes*: 73%<br>*Bacteroidetes*: 17%<br>*Proteobacteria*: 9%<br>*Actinobacteria*: < 1%
MEDIUM													

References	N=	Body weight (kg)	Age (years)	Breed	Food				Methods	Gut microbiota composition			
					Type of food	Protein (%)	Fat (%)	Fiber (%)		Stomach	Small intestine	Large intestine	Stools
Giaretta *et al.*, 2020	8	23.6	4.2 ± 1.1	N/A	N/A	N/A	N/A	N/A	FISH	N/A	N/A	Colonic surface (bacteria/microcopy area):<br>Total: 51.8 (14-687)<br>*Akkermansia spp*: 0.7 (0-78)<br>*E. coli/Shigella*: 0.23 (0-1.5)<br>*Faecalibacterium spp*: 1.02 (0.1-66.2)<br>*Helicobacter spp*: 30.2 (0-192)<br>Within crypts:<br>Total: 52.7 (0-225)<br>*Akkermansia spp*: 0.8 (0-27)<br>*E. coli/Shigella*: 0.06 (0-0.6)<br>*Faecalibacterium spp*: 0.14 (0-11.5)<br>*Helicobacter spp*: 25.65 (0-85)	N/A
Ide *et al.*, 2020	6	9.3-<br>11.4	2-6	Beagles	Commercial maintenance diet	N/A	N/A	N/A	16S rRNA gene sequencing<br><br>qPCR	N/A	N/A	N/A	Shotgun sequencing (median):<br>*Firmicutes*: 67.3%<br>*Bacteroidetes*: 4.1%<br>*Fusobacteria*: 6.9%<br>*Actinobacteria*: 4.0%<br>*Proteobacteria*: 2.2%<br>*Tenericutes*: 0%<br>*Lactobacillus:* 39.1%<br>*Bifidobacterium:* 0.6%<br>*Fusobacterium*: 7.4%<br><br>qPCR (10^{12} cells/g feces):<br>*Eubacteria*: 2<br>*Actinobacteria*: 1<br>*Bacteroidetes*: 6<br>*Fusobacteria*: 6.5<br>*Clostridium* cluster XIV: 2.5<br>*Bacteroides* spp: 1.5<br>*Bifidobacterium* spp: 1<br>*Fusobacterium* spp: 7.5<br>*Lactobacillus* spp: 2<br>*Suterella* spp: 2.2<br>*Clostridium perfringens*: 0.3<br>*Faecalibacterium prausnitzii*: 1.2
Eisenhauer *et al.*, 2019	10	13.7	4.4<br>± 0.3	Beagles	Experimental dry diet	21.9	9.8	TDF: 3.4<br>Crude fiber: 0.9<br>IF: 1.9<br>SF: 1.5	MiSeq 16S rRNA sequencing	N/A	N/A	N/A	Richness: 56.4<br>Evenness: 0.7<br>*Clostridiales*: 31.6%<br>*Bacteroidales*: 21.5%<br>*Fusobacteriales*: 22.0%<br>*Selenomonadales*: 3.8%<br>*Bifidobacteriales*: 3.4%<br>Erysipelotrichales: 3.0%
Manchester *et al.*, 2019	8	19	3<br>(1.5-6)	N/A	N/A	N/A	N/A	N/A	16S rRNA gene sequencing<br><br>qPCR	N/A	N/A	N/A	Observed species: 1243 ± 221<br>Chao1: 2533 ± 341<br>Shannon index: 5.6 ± 0.7<br>Dysbiosis index: -4<br><br>qPCR<br>*Blautia* spp: 10.2<br>*Faecalibacterium* spp: 8.3<br>*Turicibacter* spp: 6.8<br>*C. hiranonis*: 5.5<br>*Fusobacterium* spp: 9.6

References	N=	Body weight (kg)	Age (years)	Breed	Food				Methods	Gut microbiota composition			
					Type of food	Protein (%)	Fat (%)	Fiber (%)		Stomach	Small intestine	Large intestine	Stools
Minamoto *et al.*, 2019	49	17.7	4.5	N/A	N/A	N/A	N/A	N/A	Illumina 16S rRNA sequencing<br><br>qPCR	N/A	N/A	N/A	Alpha-diversity: 331 observed species<br>Chao1: 753.3<br>Shannon index: 5.2<br><br><u>qPCR (log DNA):</u><br>Total bacteria: 12.5<br>*Bacteroidetes*: 11<br>*Firmicutes*: 9<br>*Ruminococcaceae*: 7<br>*Bifidobacterium* spp: 4<br>*Blautia*: 11<br>*Faecalibacterium* spp: 6<br>*Fusobacterium*: 9<br>*Lactobacillus*: 4<br>*Streptococcus*: 4<br>*Turicibacter*: 7.5<br>*E. coli*: 6<br>*C. hiranonis*: 6<br>*C. perfringens*: 5
Nogueira *et al.*, 2019	N/A	18 ± 1.4	N/A	N/A	Experimental diet	30.8	18.5	12<br>SF: 3.2<br>IF: 8.9	Illumina 16S rRNA sequencing	N/A	N/A	N/A	*Firmicutes*: 50.6%<br>*Bacteroidetes*: 21.0%<br>*Fusobacteria*: 24.1%<br>*Proteobacteria*: 3.8%<br>*Actinobacteria*: 0.4%
Algya *et al.*, 2018	8	13	3.6 ± 0.3	Beagles	Commercial extruded dry kibble	24.1	13.3	9.6	Illumina 16S rRNA sequencing MiSeq	N/A	N/A	N/A	*Firmicutes*: 60%<br>*Bacteroidetes*: 13%<br>*Fusobacteria*: 12%<br>*Proteobacteria*: 4%<br>*Actinobacteria*: 7%
Pinna *et al.*, 2018	N/A	19.5 ± 6.2	3.6 ± 1.6	N/A	N/A	N/A	N/A	N/A	qPCR	N/A	N/A	N/A	Total bacteria (log dsDNA/g):<br>LP: 8.9<br>HP: 8.5<br>LP+FOS: 9.0<br>HP+FOS: 8.9
Schmidt *et al.*, 2018	14	13.2	N/A	N/A	Dry food and/or canned food	30.5 ± 9.6	18.2 ± 6.6	3.4 ± 1.4	Illumina 16S rRNA sequencing (V4 region)<br><br>qPCR	N/A	N/A	N/A	Chao1: 1559 ± 192<br>Shannon index: 4.8<br>Observed species: 759<br><br><u>qPCR (log DNA):</u><br>*E. coli*: 5<br>*Faecalibacterium spp*: 4.8<br>*Streptococcus spp*: 5.5<br>*Clostridium perfringens*: 4.8
Herstad *et al.*, 2017	11	19.7	4.8	English Springer Spaniel, Mixed Breads, Small Munster Lander, Eurasier, Irish Setter, English Cocker Spaniel, German Shorthaired Pointer	Commercial dry food	27.1	16.3	1.2	Illumina 16S rRNA sequencing MiSeq	N/A	N/A	N/A	Shannon index: 4.4 (3.4-5.1)<br>Observed species: 73 (49-102)<br><br>*Firmicutes*: 43%<br>*Bacteroidetes*: 22%<br>*Fusobacteria* + AK48: 28%<br>*Proteobacteria*: 5%<br>*Actinobacteria*: 1%
Masuoka *et al.*, 2017	10	N/A	2 (2)	Beagles	DS-E diet (Japan)	N/A	N/A	N/A	Illumina 16S rRNA sequencing	N/A	N/A	N/A	(log10/g feces)<br>*Bacteroidaceae*: 9.7 ± 0.3<br>*Eubacteria*: 9.4 ± 0.3<br>*Clostridia*: 8.9 ± 1<br>*Veillonella*: 4.4 ± 2<br>*Megasphaera*: 4.6 ± 1.7<br>*Lactobacilli*: 9.5 ± 0.7<br>*Enterobacteriaceae*: 7.5 ± 0.9<br>*Enterococci*: 9.2 ± 0.5<br>*Staphylococci*: 3.3 ± 0.6<br>*Bifidobacteria*: ND<br>Total bacteria: 10.3 ± 0.2

References	N=	Body weight (kg)	Age (years)	Breed	Food				Methods	Gut microbiota composition			
					Type of food	Protein (%)	Fat (%)	Fiber (%)		Stomach	Small intestine	Large intestine	Stools
Ossiprandi, 2016	13	19.6 ± 2.9	5 (3-10)	English Setter	Raw poultry meat diet	25	N/A	2	16S rRNA sequencing	N/A	N/A	N/A	*Firmicutes*: 51.2%<br>*Proteobacteria*: 27.1%<br>*Fusobacteria*: 8.5%<br>*Bacteroidetes*: 5.5%
Minamoto *et al.*, 2015	10	26.5	3.4 ± 1.6	N/A	N/A	N/A	N/A	N/A	454 pyrosequencing (V4-V6)<br><br>qPCR	N/A	N/A	N/A	Observed species: around 135<br>Shannon index: 4<br>Chao1: 390<br><br><u>qPCR (log DNA):</u><br>*Fusobacteria*: 6.5<br>*Bacteroidetes*: 8.5<br>*Ruminococcaceae*: 7.5<br>*Bifidobacterium* spp: 3.5<br>*Blautia* spp: 10<br>*Faecalibacterium* spp: 6<br>*Turicibacter* spp: 7<br>*Lactobacillus* spp: 4.8<br>*E. coli*: 3.9<br>Total bacteria: 13
Panasevich *et al.*, 2015	10	22.0 ± 2.1	6.1 ± 0.2	N/A	Experimental diet	25.1	13.5	10.8	454 pyrosequencing<br><br>qPCR	N/A	N/A	N/A	*Actinobacteria*: 0.2%<br>*Bacteroidetes*: 1.2%<br>*Firmicutes*: 56.7%<br>*Fusobacteria*: 27.4%<br>*Proteobacteria*: 1.5%<br>*Tenericutes*: 12.7%
Garcia-Mazcorro *et al.*, 2012	8	18.6 ± 2.0	0.8	Mixed breed	Commercial diet	N/A	N/A	4	FISH, qPCR, 454 pyrosequencing (V1-V3 region)	Total bacteria: 10^5 copies of 16S<br>*Proteobacteria*: 99.6% (whose 98.6% *Helicobacter*)<br>*Firmicutes*: 0.3%<br>Shannon index: 1.2 (0.9-1.6)<br>Chao1: 59 (38-81)	<u>Duodenum:</u><br>*Firmicutes*: 46.9%<br>*Proteobacteria*: 21.2%<br>*Bacteroidetes*: 6.9%	N/A	N/A
Garcia-Mazcorro *et al.*, 2011	12	21.1	0.7-10.2	10 breeds	Dry diet	N/A	N/A	N/A	16S rRNA gene pyrosequencing (V1-V3 region)	N/A	N/A	N/A	*Firmicutes*: 97.5%<br>*Bacteroidetes*: 0.1-11%<br>*Fusobacteria*: 0.1-0.8%<br>*Proteobacteria*: 0.1%<br>*Actinobacteria*: 0.9-2%
Handl *et al.*, 2011	12	N/A	4 (0.7-10.2)	Labrador Retriever Mix, Maltese Poodle Mix; Blue Heeler, Labrador Retriever, Miniature Dachshund, Rhodesian Ridgeback Mix, Jack Russel Terrier Mix, Pembroke Welsh Corgi, Mongrel, Boxer, Chesapeake Bay Retriever, Labrador Retriever	Commercial diet	N/A	N/A	N/A	16S rRNA pyrosequencing	N/A	N/A	N/A	*Clostridiales*: 65%<br>*Lactobacillales*: 17%<br>*Erysielotrichales*: 13%<br>*Bacteroidales*: 2.5%<br>*Coriobacteriales*: 2.5%<br>Others: < 1%<br><br>*Fungi*: 99.6% of fungi sequences are *Ascomycota* (whose 85.5% *Saccharomyces*)
Swanson *et al.*, 2011	6	20.3	1.7	Hound-cross	Experimental diet	30	20	1.4	Whole genome pyrosequencing	N/A	N/A	N/A	*Firmicutes*: 31-35%<br>*Bacteroidetes*: 37-38%<br>*Fusobacteria*: 7-9%<br>*Actinobacteria*: 1%<br>*Proteobacteria*: 13-15%
Middelbos *et al.*, 2010	6	20.3	1.7	Mongrels, Hound-crosses	Experimental diet	30	20	1.4	16S rRNA gene pyrosequencing (V3 region)	N/A	N/A	N/A	*Firmicutes*: 15-28%<br>*Bacteroidetes*: 32-34%<br>*Fusobacteria*: 24-40%<br>*Actinobacteria*: 0.8-1.4%<br>*Proteobacteria*: 5-6%

References	N=	Body weight (kg)	Age (years)	Breed	Food				Methods	Gut microbiota composition			
					Type of food	Protein (%)	Fat (%)	Fiber (%)		Stomach	Small intestine	Large intestine	Stools
Suchodolski, Camacho, & Steiner, 2008a	6	N/A	6.2<br>(3.6-7)	Hound dog	N/A	N/A	N/A	N/A	16S rRNA gene pyrosequencing (V1-V3 region)<br><br>Duodenum. jejunum. ileum and colon contents	N/A	Duodenum:<br>*Clostridiales*: 40%<br>*Lactobacillales*: 24%<br>*Fusobacteriales*: 3%<br>*Enterobacteriales*: 33%<br>Jejunum:<br>*Clostridiales*: 40%<br>*Lactobacillales*: 11%<br>*Fusobacteriales*: 14%<br>*Enterobacteriales*: 28%<br>*Bacteroidales*: 7%<br>Ileum:<br>*Clostridiales*: 23%<br>*Lactobacillales*: 1%<br>*Fusobacteriales*: 31%<br>*Enterobacteriales*: 22%<br>*Bacteroidales*: 23%	*Clostridiales*: 25%<br>*Lactobacillales*: 12%<br>*Fusobacteriales*: 29%<br>*Enterobacteriales*: 1%<br>*Bacteroidales*: 33%	N/A
Xenoulis *et al.*, 2008	9	N/A	5<br>(2.7-6)	Greyhounds, Beagles	N/A	N/A	N/A	N/A	16S rRNA pyrosequencing	N/A	Duodenum biopsies:<br>*Firmicutes*: 46.6%<br>*Proteobacteria*: 26.6%<br>*Bacteroidetes*: 11.2%<br>*Spirochaetes*: 10.3%<br>*Fusobacteria*: 3.6%<br>*Actinobacteria*: 1%<br>Shannon index: 4.1 (3.4-4.6)<br>Chao1: 324 (274-470)<br><br>Order:<br>*Clostridiales*: 19.6%<br>*Lactobacillales*: 14.1%<br>*Bacillales*: 12%	N/A	N/A
Mentula *et al.*, 2005	22	N/A	1-3	Beagles	Canned food (Pedigree)	N/A	N/A	N/A	Bacterial culture	N/A	Jejunal chyme:<br>Total counts: 4 x 10^4 CFU/g	N/A	Total counts: 3 x 10^{10} CFU/g
Flickinger *et al.*, 2003	4	12	3	Beagles	Commercial	N/A	N/A	N/A	N/A	N/A	N/A	N/A	Total anaerobe:<br>10.4 log10 CFU/g<br>Total aerobes: 10.2<br>*Bifidobacteria*: 9.4
**LARGE**													
Sandri *et al.*, 2016	8	30.3	4.2 ± 2.8	Boxer	Commercial dry food 358 kcal/100g	26.7	10.6	2.8	16S rRNA (V3-V4 region)<br><br>Illumina 16S rRNA sequencing	N/A	N/A	N/A	Shannon index: 2.9 ± 0.3<br>*Actinobacteria*: 1.0%<br>*Bacteroidetes*: 22.1%<br>*Firmicutes*: 70.5%<br>*Fusobacteria*: 4.7%<br>*Proteobacteria*: 1.3%
**UNCLASSIFIED**													
Van den Abbeele *et al.*, 2020	3	N/A	N/A	N/A	N/A	N/A	N/A	N/A	qPCR	N/A	N/A	N/A	*Firmicutes*: 40%<br>*Bacteroidetes*: 35%<br>*Actinobacteria*: 4%<br>*Fusobacteria*: 20%<br>*Proteobacteria*: 1%
Alessandri *et al.*, 2019	141	22.2<br>(1-62)	4.2<br>(0.1-13)	51 breeds	Commercial dry food	N/A	N/A	N/A	Shotgun metagenomic	N/A	N/A	N/A	*Bacteroidetes*: 37.7%<br>*Fusobacteria*: 24.3%<br>*Firmicutes*: 28.5%<br>*Proteobacteria*: 8.1%<br>*Actinobacteria*: 1.0%<br>*Tenericutes*: 0.2%<br><br>Genus:<br>*Fusobacterium*: 24.1%<br>*Bifidobacterium:* 0.2%<br>*Prevotella* 9: 15.3%<br>*Bacteroides*: 14.1%<br>*Faecalibacterium*: 5.6%<br>*Suterella*: 3.1%

References	N=	Body weight (kg)	Age (years)	Breed	Food				Methods	Gut microbiota composition			
					Type of food	Protein (%)	Fat (%)	Fiber (%)		Stomach	Small intestine	Large intestine	Stools
Blake *et al.*, 2019	34	N/A	4 (1-12)	Mixed Breed, German Shepherd dog, Miniature Schnauzer, Mastiff, Bull Terrier, Chinese Crested Dog, Dachshund, English Cocker Spaniel, Husky, Miniature Poodle, Vizsla, Weimaraner	Maintenance diet	N/A	N/A	N/A	qPCR	N/A	N/A	N/A	*Faecalibacterium*: 6.6<br>*Turicibacter*: 5.3<br>*Streptococcus*: 3.8<br>*E. coli*: 4.4<br>*Blautia*: 9.7<br>*Fusobacterium*: 8.7<br>*Clostridium hiranonis*: 6.2<br>*Lactobacillus*: 3.3<br>*Bifidobacterium*: 3.5<br>*Enterococcus*: 2.7<br>Dysbiosis index: -3.2
Bresciani *et al.*, 2018	11	N/A	4 (1-13)	Mixed Breeds, Golden Retrievers, Newfoundland, Bernese Mountain Dog, Border Collie, Beagle, Australian Shepherd Dog, English Setter	N/A	N/A	N/A	N/A	Illumina 16S rRNA sequencing (V4 region)<br><br>qPCR	N/A	N/A	N/A	Chao1: 1528<br>Shannon index: 5.2<br><br><u>qPCR (log DNA):</u><br>Total: 11.5<br>*Fusobacterium*: 8.8<br>*Faecalibacterium*: 7<br>*Hiranonis*: 6.5<br>*Turicibacter*: 7<br>*Blautia*: 9.5<br>*Streptococcus*: 7.5<br>*E. coli*: 6.5
Herstad *et al.*, 2018	6	N/A	4-10	Mixed Breed	Commercial dry food	N/A	N/A	N/A	Illumina 16S rRNA sequencing	N/A	N/A	N/A	*Firmicutes*: 68 ± 14%<br>*Bacteroidetes*: 16 ± 11%<br>*Proteobacteria*: 2 ± 3%<br>*Actinobacteria*: 4 ± 3%<br><br><u>Genus:</u><br>*Megamonas*: 14 ± 18%<br>*Prevotella*: 9 ± 9%<br>*Bacteroides*: 2 ± 1%<br>*Fusobacterium*: 9 ± 12%<br>*Blautia*: 10 ± 6%<br>*Clostridium* XI: 15 ± 12%<br>*Faecalibacterium*: 6 ± 4%
Park *et al.*, 2018	15	N/A	1-13	Bulldog, Welsh corgi, Dachshund, Yorkshire terrier, silky terrier, Poodle, shih tzu, Labrador retriever, Spitz, cocker spaniel and miniature pinscher	N/A	N/A	N/A	N/A	Plate count<br><br>Illumina 16S rRNA sequencing	N/A	N/A	N/A	(log10 bacteria/g feces)<br>*Enterobacteriaceae*: 9.2 ± 0.4<br>*Enterococcus*: 9.4 ± 1.2<br>*Lactobacillus*: 9.0 ± 0.7<br>*Eubacterium*: 9.1 ± 0 (found in 20% dogs)<br>*Bacteroidaceae*: 9.0 ± 0.5<br>*Clostridium* spp: 3.8 ± 2.5 (60% of dogs)<br>Total: 10.0 ± 0.5<br>No yeast or *Bifidobacterium* detected
Salas-Mani *et al.*, 2018	19	N/A	5 (2-15)	Beagles	N/A	30.9	8.4	TDF: 24.8<br>CF: 9.8<br>SF: 2.6<br>IF: 22.2	Illumina 16S rRNA sequencing	N/A	N/A	N/A	*Firmicutes*: 69.4%<br>*Bacteroidetes*: 16.7%<br>*Fusobacteria*: 10.2%<br>*Actinobacteria*: 3.3%<br>*Proteobacteria*: 1.4<br><br>*Clostridia*: 33.2%<br>*Bacilli*: 2.6%<br>*Erypelotrichi*: 32.5%<br>*Faecalibacterium*: 3.0%
Xu *et al.*, 2016	16	N/A	N/A	N/A	N/A	N/A	N/A	N/A	qPCR	N/A	N/A	N/A	Total: 10.5 log10 copies 16S/g of fresh stool<br>*Firmicutes*: 110%<br>*Bacteroidetes*: 35.5%<br>*Enterobacteriaceae*: 0.6%<br>*Lactobacillus*: 8.7%<br>*Clostridial* cluster I: 13.1%

References	N=	Body weight (kg)	Age (years)	Breed	Food				Methods	Gut microbiota composition			
					Type of food	Protein (%)	Fat (%)	Fiber (%)		Stomach	Small intestine	Large intestine	Stools
Deng & Swanson, 2015	N/A	N/A	N/A	N/A	N/A	N/A	N/A	N/A	N/A	N/A	N/A	N/A	<u>*Fungi*:</u><br>*Ascomycota*: 97.9%<br>*Basidiomycota*: 1%<br><br><u>*Archaea*:</u> around 1.1% (mainly methanogens)
Handl *et al.*, 2013	10	N/A	4.2 ± 2.9	N/A	Commercial diet	N/A	N/A	N/A	454 pyrosequencing<br><br>qPCR	N/A	N/A	N/A	Shannon index: 1.1<br>Evenness: 0.4<br>*Firmicutes*: > 90%<br>*Actinobacteria*: 1.7 ± 2.9%<br>*Fusobacteria*: 6.4 ± 19.1%<br><u>Ordre:</u><br>*Clostridiales*: 67.8 ± 35%<br>*Bacillales*: 15.4 ± 19.1%
Hooda *et al.*, 2012	N/A	N/A	N/A	N/A	N/A	N/A	N/A	N/A	N/A	Mixture in aerobe and anaerobe bacteria. mainly Gram positive. As in humans. *Helicobacter* spp are presents and could be pathogens	<u>Duodenum et Jejunum:</u><br>Predominance of *Eubacterium*, *Bacteroides*, *Clostridium*, *Fusobacterium*, *Bifidobacterium* and *Lactobacillus* spp<br><u>Ileum:</u><br>*Bacteroides*, *Eubacterium*, *Fusobacterium*, *Clostridium*, *Lactobacillus* and *Bifidobacterium* predominate among the anaerobe bacteria<br>There is also aerobe and facultative anaerobe bacteria: *Streptococcus*, *Staphylococcus*, *Pasteurella*, *Escherichia* and *Enterobacter*	High density and diversity, Predominant bacteria are from *Bacteroides*, *Bifidobacterium*, *Fusobacterium*, *Peptostreptococcus*, *Eubacterium*, *Clostridium*, *Peptococcus* and *Lactobacillus*	N/A
Handl *et al.*, 2011	12	N/A	0.7-10.2	10 breeds	N/A	N/A	N/A	N/A	16S rRNA gene pyrosequencing (V1-V3 region)	N/A	N/A	N/A	*Actinobacteria*: 1.8%<br>*Bacteroidetes*: 2.2%<br>*Firmicutes*: 95%<br>*Fusobacteria*: 0.3%
Suchodolski, 2011	N/A	N/A	N/A	N/A	N/A	N/A	N/A	N/A	Review (or unpublished data)	N/A	16S rRNA gene analysis:<br>2 archaeal phyla: *Crenarchaeota* et *Euryarchaeota.*<br>Most abundant class: Methanobacteria (*Methanosphaera* et *Methanobrevibacter*)	N/A	<u>454 pyrosequencing:</u><br>4 fungal phyla with 90% *Ascomycota*<br>> 5% *Neocallimastigomycota*<br><br><u>Metagenomic sequencing:</u><br>Archaea represents 1.1% of total microbiota (methanogens are the most abundant)<br><br>Viruses are mainly bacteriophages and dsDNA viruses represent 0.4% of detected sequences
Jia *et al.*, 2010	8	N/A	4-13	N/A	Commercial diet	18.2	9.9	CF: 1.6<br>SF: 5.8<br>IF: 1.2	FISH<br><br>DGGE	N/A	N/A	N/A	*Firmicutes*: 31%<br>*Actinobacteria*: 12%<br>*Bacteroidetes*: <1% (10^8 cells/g feces)

References	N=	Body weight (kg)	Age (years)	Breed	Food				Methods	Gut microbiota composition			
					Type of food	Protein (%)	Fat (%)	Fiber (%)		Stomach	Small intestine	Large intestine	Stools
Suchodolski *et al.*, 2009		N/A	N/A	N/A	N/A	N/A	N/A	N/A	16S rRNA pyrosequencing	N/A	Jejunum mucosa: *Proteobacteria*: 46.7% *Firmicutes*: 15% *Actinobacteria*: 11.2% *Spirochaetes*: 14.2% *Bacteroidetes*: 6.2% *Fusobacteria*: 5.4% *Tenericutes*, *Cyanobacteria*, Verrucomicrobia and Chloroflexi: < 0.1% *Gammaproteobacteria*: 40% *Spirochaetes*: 13% *Actinobacteria*: 12% *Clostridia*: 10% *Bacteroides*: 6% *Fusobacteria*: 4% *Alphaproteobacteria*: 3%	N/A	N/A
Biagi, Cipollini, & Zaghini, 2008	8	N/A	N/A	N/A	N/A	N/A	N/A	N/A	Plate count	N/A	N/A	N/A	(Log CFU/mL) *Lactobacilli*: 7.9 Coliforms: 7.7 *Enterococci*: 7.5
Suchodolski *et al.*, 2008b	64	N/A	N/A	N/A	N/A	N/A	N/A	N/A	Illumina 16S rRNA gene sequencing	N/A	*Fungi* DNA detected in 60.9% of dogs and significatively increased in mucosal samples (82.8%) compared to luminal samples (42.9%): mainly *Ascomycota* and *Basidiomycota*. 3 major classes: *Saccharomycetes*, *Dothideomycetes* and *Hymenomycetes*.	N/A	N/A

<u>Supplementary Table S6</u>: Overview of studies reporting short chain fatty acids and other metabolites production of healthy adult dogs depending on body weight, measured in faeces.

References are organized according to dog sizes (small, medium, large, unclassified) in reverse chronological order. BCFA: branched chain fatty acids; DM: dry matter; GC: gas chromatography; GC/MS: gas spectrometry coupled with mass spectrometry; HP: high protein; HPLC: high performance liquid chromatography; LC: liquid chromatography; LP: low protein; N/A: not applicable; SCFA: short chain fatty acid.

References	N=	Weight (kg)	Age (years)	Breeds	Food				Methods	Units	Fatty acids								Other metabolites		
					Type of food	Protein (%)	Fat (%)	Fiber (%)			Total SCFA	Acetate	Propionate	Butyrate	Total BCFA	Isobutyrate	Isovalerate	Valerate	Phenol	Indole	Ammonia
**SMALL**																					
Igarashi *et al.*, 2017	25	6.1	8.6 (2-16.4)	N/A	Low protein (LP), high protein (HP)	N/A	N/A	N/A	HPLC	µmol/g	N/A	LP: 92.8 HP: 93	LP: 58.3 HP: 41.9	N/A	LP: 176 HP: 159	N/A	N/A	N/A	N/A	N/A	LP: 39.1 HP: 54.1
Beloshapka *et al.*, 2014	12	8.4 (1-2)	3.3 (1-6)	Miniature Schnauzer	Maintenance diet	36.1	25.4	8.86 SF: 4.46 IF: 4.4	GC	mmol/g	453.17	247.4	164.9	41.4	25.1	9.7	14.7	0.7	1.1	2.8	1035.4
Goudez *et al.*, 2011	7	4-6.5	N/A	Miniature Poodle, Jack Russell, Miniature Schnauzer	Experimental diet	28.6	17.6	7.8	GC	µmol/g	306.6 ± 22.3	N/A	N/A	N/A	17.1 ± 2.5	N/A	N/A	N/A	0.0	1.6 ± 0.6	0.9 ± 0.2 mg/g
Weber *et al.*, 2004	6	3.5	1.25	Miniature Poodle	Experimental dry food	39.2	16.3	9.8	GC	Mmol/kg DM	448 ± 7	N/A	N/A	N/A	N/A	N/A	N/A	N/A	N/A	N/A	N/A
**MEDIUM**																					
Ide *et al.*, 2020	6	9.3-11.4	2-6	Beagles	Maintenance diet	N/A	N/A	N/A	HPLC	µmol/g	N/A	88	63	9.65 ± 1	N/A	N/A	N/A	N/A	N/A	N/A	N/A
Alexander *et al.*, 2019	12	10.35 ± 0.88	3.17± 0.82	Beagles	Experimental diet	38.6	17.3	7.2	GC	µmol/g	414.1	229.9	122.0	62.2	31.6	11.3	18.3	1.9	1.5	3.1	304.5
Detweiler *et al.*, 2019	8	12.8 ± 1.7	4.6 ± 0.6	Beagles	Experimental diet	31.7	11.9	SF: 2.6 IF: 2.4	GC	µmol/g	267	150.8	82.6	33.5	22.9	8.8	13.3	0.8	0.9	2.2	152.2
Eisenhauer *et al.*, 2019	10	13.7	4.4 ± 0.3	Beagles	Experimental dry diet	21.9	9.8	TDF: 3.4 Crude fiber: 0.9 IF: 1.9 SF: 1.5	GC	µmol/g	91.1	44.6	18.6	20.1	4.7	2.0	2.6	3.3	34.6	702	27.7
Minamoto *et al.*, 2019	49	17.7	4.5	N/A	N/A	N/A	N/A	N/A	GC/MS	µmol/g	377	224	106	28.9	N/A	N/A	N/A	N/A	N/A	N/A	N/A
Nogueira *et al.*, 2019		18 ± 1.38	N/A	N/A	Experimental diet	30.8	18.5	12 SF: 3.2 IF 8.9	N/A	µmol/g	341.6	205.5	100.8	35.3	18.1	7.6	11.2	0.6	1.2	2.4	131.2
Algya *et al.*, 2018	8	13	3.57± 0.29	Beagles	Commercial extruded dry kibble	24.1	13.3	9.6	N/A	µmol/g	417.1 ± 4	257.5 ± 224	116 ± 16	43.6 ± 8	N/A	5.3	9.7	0.7	0.3	1.2	26
(Pinna *et al.* 2018	3.6 ± 1.6	19.5 ± 6.2	N/A	N/A	High protein diet	N/A	N/A	N/A	HPLC	µmol/g	N/A	93	41.9	19	N/A	2.5	4.7	N/A	N/A	N/A	54.1
Herstad *et al.* 2017	11	19.65	4.77	English Springer Spaniel, Mixed Breeds, Small Munster Lander, Eurasian, Irish Setter, English Cocker Spaniel, German Shorthaired Pointer	Commercial dry food	27.1	16.3	1.2	GC	%	N/A	53.2 (50.8-58.3)	32.8 (29.4-37)	11.1 (8-13)	N/A	N/A	3.6 (1.3-4.4)	N/A	N/A	N/A	N/A

References	N=	Weight (kg)	Age (years)	Breeds	Food				Methods	Units	Fatty acids								Other metabolites		
					Type of food	Protein (%)	Fat (%)	Fiber (%)			Total SCFA	Acetate	Propionate	Butyrate	Total BCFA	Isobutyrate	Isovalerate	Valerate	Phenol	Indole	Ammonia
Beloshapka *et al.* 2016)	48	10	3.4	Beagles	Experimental diet	30.1	18.6	5.7 SF: 1.5 IF: 4.2	GC	µmol/g	334.5	187.2	96.1	51.3	25.2	9.6	15	0.6	2.5	2.7	140.1
Wambacq *et al.* 2016	8	11.6	5-5.5	Beagles	Experimental diet	16.6	14.6	1.8 SF: 2 IF: 4.7	GC	mmol/day	SF: 14.6 IF: 9.0	SF: 8.1 IF: 5.0	SF: 4.9 IF: 2.6	SF: 1.1 IF: 0.9	N/A	SF: 0.35 IF: 0.35	SF: 0.4 IF: 0.4	SF: 0.03 IF: 0.03	N/A	N/A	SF: 30.7 IF: 31.8
Hang *et al.* 2013	5	19	5	Beagles	Commercial diet	N/A	N/A	N/A	N/A	mM	196 (165-221)	118 (90-135)	49.3 (45.3-55.9)	21.3 (14.8-27.8)	N/A	N/A	N/A	N/A	N/A	N/A	N/A
Beloshapka, Wolff, & Swanson, 2012	8	20 ± 0.5	3.5 ± 0.5	Mixed Breed	Experimental diet	35.2	20.2	8.6	GC	µmol/g	423.3	279.5	93.8	50	29.6	12.1	16.7	0.8	1.6	3.2	904.1
Goudez *et al.*, 2011	6	14.4	4.8 ± 0.5	Standard Schnauzer	Experimental diet	28.6	17.6	7.8	GC	µmol/g	392.2 ± 39	N/A	N/A	N/A	19.3 ± 0.9	N/A	N/A	N/A	0	1.3	1.1
	6	27.2		Giant Schnauzer							364 ± 24.4	N/A	N/A	N/A	22.2 ± 1.0	N/A	N/A	N/A	0	0.6	0.7
	8	23.1		German Shepherd							412.5 ± 23.3	N/A	N/A	N/A	22.1 ± 2.1	N/A	N/A	N/A	0.3	1.7	1.5
Faber *et al.*, 2011	4	22 ± 2.1	3.4	N/A	N/A	31.6	21.6	11.5	HPLC	µmol/g	339.4	209.3	84.1	46	16.1	5.9	9.2	1	112 µg/g	133.5 µg/g	2.3 mg/g
Weber *et al.*, 2004	6	12.6	1.25	Standard Schnauzer	Experimental dry food (Royal canin)	39.2	16.3	9.8	GC	mmol/kg	793 ± 100	N/A	N/A	N/A	N/A	N/A	N/A	N/A	N/A	N/A	N/A
	6	23.3	1.25	Giant Schnauzer							996 ± 61	N/A	N/A	N/A	N/A	N/A	N/A	N/A	N/A	N/A	N/A
Zentek *et al.*, 2004	8	12.4 ± 2.2	1-11	Beagles	Commercial diet	31.0	14.6	2.5	GC	µmol/g	256	63.4%	22.8%	9.5%	N/A	1.7	2.5	0.19	N/A	N/A	87
Flickinger *et al.* 2003	4	12	3	Beagles	Commercial	N/A	N/A	N/A	LC	mmol/g	63.1	30.8	20.3	5.4	2.5	0.5	0.6	1.4	0.02 mg/g	0.1 mg/g	4.4 mg/g
**LARGE**																					
Sandri *et al.* 2016	8	30.3	4.2 ± 2.8	Boxer	Commercial dry food	26.7	10.6	2.8	HPLC	mmol/g	0.9	0.4	0.4	0.2	N/A	N/A	N/A	N/A	N/A	N/A	N/A
Cutrignelli *et al.*, 2009	2	32.5	3	German Shepherd	Commercial dry food	25.8	N/A	6.6	GC	mmol/mL	34.6	20.7	11.2	2.7	N/A	1.6	0.9	1.6	N/A	N/A	N/A
	2	59.8	3	Neapolitans Mastiff	Commercial dry food	25.8	N/A	6.6			45.9	22.3	20.2	3.4	N/A	0.6	0.5	0.5	N/A	N/A	N/A
Weber *et al.*, 2004	6	46.3	1.25	Great Dane	Experimental dry food	39.2	16.3	9.8	GC	mmol/kg	1184 ± 259	N/A	N/A	N/A	N/A	N/A	N/A	N/A	N/A	N/A	N/A
**UNCLASSIFIED**																					
Blake *et al.*, 2019	34	N/A	4 (1-12)	Mixed Breed, German Shepherd dog, Miniature Schnauzer, Mastiff, Bull Terrier, Chinese Crested dog, Dachshund, English Cocker Spaniel, Husky, Miniature Poodle, Vizsla, Weimaraner	Maintenance diet	N/A	N/A	N/A	Spectro-photometric analysis	µmol/g	249.9 (122.9-501.6)	136.7 (43.7-334.2)	50 (0-137.4)	9.6 (0-39.3)	N/A	0	0	0	N/A	N/A	N/A
Xu *et al.*, 2016	16	N/A	N/A	N/A	N/A	N/A	N/A	N/A	GC	%	123.9 ± 36.9 µmol/g	63 ±11.6	26.3 ± 10.5	7 ±3.7	N/A	1.5	1.8	N/A	N/A	N/A	324 ±195 µmol/g
(Biagi *et al.* 2008)	8	N/A	N/A	N/A	N/A	N/A	N/A	N/A	N/A	mM	20.8	10.9	0.8	0.4	N/A	N/A	N/A	N/A	N/A	N/A	36.9
(Propst *et al.* 2003)	7	N/A	« adult »	N/A	Kibble	32.7	23.5	N/A	GC	µmol/g	406.4	274.6	92.7	39.2	43.4	12.6	12.6	18.1	3.0	20.0	222.5
